# Casein kinase 2-mediated phosphorylation of the splicing factor SF3B3 plays a key role in esophageal squamous cell carcinoma progression

**DOI:** 10.1371/journal.pbio.3003729

**Published:** 2026-04-10

**Authors:** Du-chuang Wang, Jia-yuan Li, Xiao-bing Wang, Guo-sheng Hu, Rui-chao Nie, Bin Zheng, Yao-hui He, Wen Liu

**Affiliations:** 1 State Key Laboratory of Cellular Stress Biology, School of Life Sciences, Faculty of Medicine and Life Sciences, Xiamen University, Xiamen, Fujian, China; 2 Fujian Provincial Key Laboratory of Innovative Drug Target Research, School of Pharmaceutical Sciences, Faculty of Medicine and Life Sciences, Xiamen University, Xiamen, Fujian, China; 3 Jiangxi Health Industry Institute of Traditional Chinese Medicine, Nanchang, Jiangxi, China; 4 National Institute for Data Science in Health and Medicine, Xiamen University, Xiang’an South Road, Xiamen, Fujian, China; 5 Department of Thoracic Surgery, Fujian Medical University Union Hospital, Fuzhou, Fujian, China; 6 Institute for Future Sciences, University of South China, Changsha, Hunan, China; 7 Shenzhen Research Institute of Xiamen University, Shenzhen, Guangdong, China; Consejo Nacional de Investigaciones Científicas y Técnicas: Consejo Nacional de Investigaciones Cientificas y Tecnicas, ARGENTINA

## Abstract

Protein kinases play a crucial role in regulating cellular processes, and their dysregulation is frequently implicated in various diseases, including cancer. Targeting protein kinases represents a promising therapeutic strategy for cancer treatment. Esophageal squamous cell carcinoma (ESCC) constitutes over 90% of esophageal cancer cases in high-incidence regions, with a global five-year survival rate below 20%. Here, we report that CK2 is aberrantly activated in ESCC, identified through kinase-substrate enrichment analysis (KSEA) of large-scale proteomic and phosphoproteomic data. Functional enrichment revealed the splicing factor SF3B3 as a clinically relevant CK2 substrate. We demonstrated that CK2-mediated phosphorylation of SF3B3 T1200 plays a pivotal role in ESCC progression. Mechanistically, CK2-mediated phosphorylation of SF3B3 enhances its affinity for the deubiquitinase USP7, leading to SF3B3 deubiquitination and subsequent protein stabilization. This stabilization drives ESCC progression by regulating alternative splicing (AS) events, including a critical event involving the inclusion of exon 4 in the *EXOSC2* transcript. Furthermore, we demonstrated that SF3B3 T1200 phosphorylation specifically facilitates its incorporation into the U2 snRNP complex, directly promoting the aforementioned *EXOSC2* exon 4 inclusion. Crucially, targeting CK2 or USP7, either individually or in combination, effectively suppressed ESCC progression. Our findings uncover a key molecular mechanism underlying SF3B3 stabilization and AS regulation, offering novel therapeutic opportunities for ESCC.

## Abstract 2

Protein kinases play a crucial role in regulating cellular processes, and their dysregulation is frequently implicated in various diseases, including cancer. This study shows that casein kinase 2-mediated phosphorylation of the splicing factor SF3B2 enhances its deuquitination and stability to drive aberrant alternative splicing and promote ESCC progression.

## Introduction

Esophageal cancer is one of the most common malignant tumors of the digestive tract worldwide, ranking 11th in terms of new cases and sixth in terms of mortality among all cancers [1]. It claims the lives of over 440,000 people annually [2]. In China, esophageal squamous cell carcinoma (ESCC) constitutes more than 90% of esophageal cancer cases, with a five-year survival rate below 20% [3]. The lack of early symptoms often results in diagnoses at advanced stage, thereby limiting therapeutic options.

Post-translational modifications of proteins are crucial for regulating cellular physiology and are involved in various biological processes, including signal transduction, transcriptional regulation, protein homeostasis, protein localization, and phase separation [4]. PTMs such as phosphorylation, acetylation, methylation, ubiquitination, SUMOylation, NEDDylation, glycosylation, palmitoylation, and S-nitrosylation intricately regulate cellular functions [4]. Protein phosphorylation is one of the most prevalent and dynamic PTMs, regulated by kinases and phosphatases, and it plays a key role in regulating cellular signal transduction, protein-protein interactions, gene transcription, among many other essential functions [4,5]. For example, SRC-induced phosphorylation of ANXA2 at tyrosine 23 leads to its nuclear translocation and enhances the metastatic potential of ESCC cells [6]. PRKD2-mediated phosphorylation of DSG2 at T730 promotes ESCC progression, and targeting this phosphorylation site on DSG2 represents a potential therapeutic approach for ESCC [7]. Ubiquitination is another widely prevalent PTM that is tightly regulated by a cascade of enzymes, including E1, E2, and E3 ubiquitin ligases, as well as deubiquitinating enzymes (DUBs) [8]. Dysregulation of ubiquitination is implicated in various diseases, including cancer, neurodegenerative disorders, and autoimmune diseases [8,9]. For instance, the E3 ubiquitin ligase PARK2 inhibits ESCC progression by promoting YAP degradation through K48-linked ubiquitination at lysine 90 [10], while the DUB USP4 stabilizes TAK1 protein levels via deubiquitination, thereby promoting ESCC progression [11].

Protein kinases, as catalysts of phosphorylation reactions, are frequently dysregulated in various human diseases, particularly cancer [12]. Protein kinase CK2, a highly conserved serine/threonine kinase comprising two catalytic (α or α′) and two regulatory (β) subunits, is frequently over-activated in numerous tumors [13,14]. As a constitutively active enzyme with a broad substrate range, CK2 is implicated in a diverse array of cellular processes, including cell proliferation, apoptosis, and DNA damage repair, promoting tumorigenesis through the phosphorylation of various substrates such as AKT, P53, and STAT3 [15–17]. Previous studies have reported that Casein kinase II subunit α (CSNK2A1) was nominated as a potential kinase target for ESCC through integrated kinase-substrate network analysis [18]. Given its central role in cellular signaling and its frequent dysregulation in cancer, CK2 has emerged as a promising therapeutic target [19,20]. However, the role of CK2 in ESCC remains insufficiently understood, and its potential involvement in ESCC tumorigenesis warrants further investigation.

Splicing is a crucial cellular process involving the removal of introns and the ligation of exons from pre-mRNA to generate mature mRNA [21,22]. Cancer cells often exhibit aberrant splicing patterns compared to normal cells, contributing to tumorigenesis. These splicing alterations may be caused by mutations in splicing regulatory elements, or dysregulated splicing factors due to abnormal expression, mutations, or post-translational modifications [21,22]. Dysregulated alternative splicing (AS) has been implicated in cancer initiation and progression and is increasingly recognized as a potential therapeutic target [23,24]. For example, the long isoform of BOLA3 (BOLA3-L), which includes exon 3, is highly expressed in ESCC and correlates with poor prognosis [25]. Similarly, LOXL2Δ72 significantly promotes ESCC cell migration and invasion [26].

The SF3b complex, an essential component of the U2 small nuclear ribonucleoprotein (U2 snRNP) complex, comprises seven subunits: SF3B1, SF3B2, SF3B3, SF3B4, SF3B5, SF3B6, and PHF5A. This complex plays a critical role in branch point sequence recognition during the early stages of spliceosome assembly [27–29]. SF3B1 primarily participates in RNA recognition, binding, and splice sites selection, while SF3B3 mainly functions as a scaffold protein, maintaining the complex’s structural stability and assisting in the assembly of other subunits [28]. Recurrent mutations in SF3B1 are frequently observed across a range of cancers, causing splicing alterations [30,31]. Previous studies have reported that the upregulated long noncoding RNA LINC02820 interacts with SF3B3 and cooperates with TNFα to amplify the NF-κB signaling pathway, thereby promoting ESCC metastasis [32]. While the functional roles of SF3B1, SF3B4, and PHF5A have been extensively studied in various cancers, including ESCC [30,33,34], the involvement of other SF3b complex members in ESCC progression remains poorly understood.

In this study, we performed kinase-substrate enrichment analysis (KSEA) utilized large-scale proteomic and phosphoproteomic data from ESCC, which we previously reported [1]. Our analysis revealed aberrant activation of CK2 in ESCC [1,35]. Additionally, we observed that multiple members of the SF3b complex are highly phosphorylated in ESCC. We identified Thr1200 as a CK2 phosphorylation site on SF3B3 and demonstrated that phosphorylation of SF3B3 at T1200 stabilizes the protein, thereby promoting ESCC cell proliferation, migration, and invasion. Mechanistically, phosphorylation of SF3B3 at T1200 enhances its interaction with deubiquitinase USP7, which removes K48-linked ubiquitination on SF3B3, thereby preventing its degradation. Furthermore, utilizing third-generation sequencing, we systematically characterized the splicing landscape regulated by SF3B3 in ESCC, revealing that SF3B3 regulates numerous AS events, which are predominantly enriched in mRNA metabolic processes. Functional analysis demonstrated that the impact of SF3B3 on ESCC malignant behaviors is partly mediated through the long isoform of EXOSC2 (EXOSC2-L) induced by SF3B3. Finally, we demonstrated that targeting CK2 and USP7, either individually or in combination, effectively suppresses ESCC progression.

## Results

### Elevated CK2 activity and CK2-mediated SF3B3 phosphorylation are clinically relevant in ESCC patients

**Fig 1:**
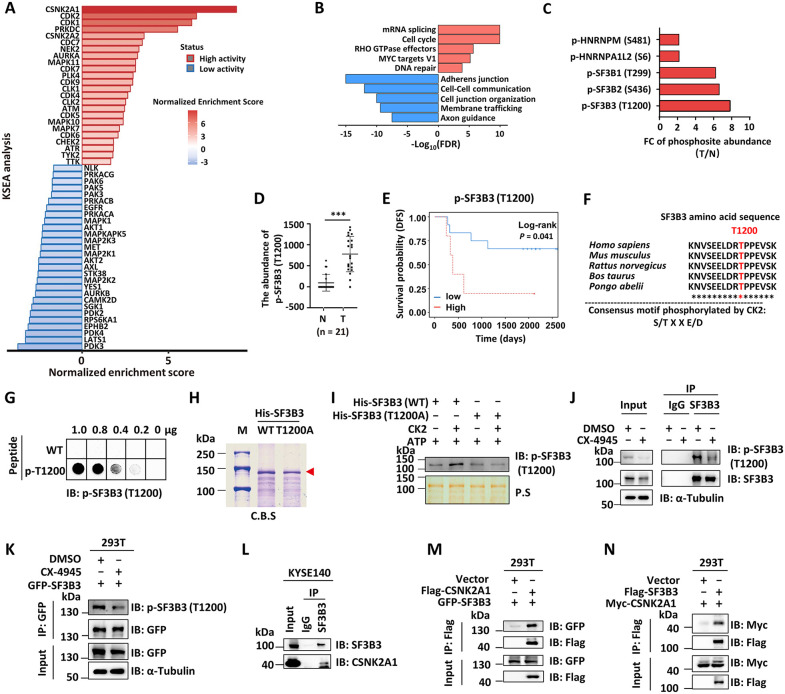
Elevated CK2 activity and CK2-mediated SF3B3 phosphorylation are clinically relevant in ESCC patients. **(A)** Kinase-substrate enrichment analysis (KSEA) was performed based on phosphoproteomic data in ESCC. Kinases are considered hyper-activated when enrichment score > 0 (red) and *P* < 0.05, while they are regarded as inhibited when the enrichment score < 0 (blue) and *P* < 0.05. **(B)** KEGG pathway enrichment analysis results are shown for differential phosphoproteins. Pathways enriched in the upregulated (*n* = 1,038) and down-regulated (*n* = 574) phosphoproteins are indicated by pink and blue bars, respectively. **(C)** Fold change (FC) in the abundance of phosphosites conforming to the CK2 consensus motif (S/T X X E/D) is shown (BH adjusted *P* value < 0.01) **(D)** The abundance of p-SF3B3 (T1200) in ESCC tumor (T) and adjacent normal (N) tissues (*n* = 21). **(E)** Kaplan–Meier plots of Disease-Free Survival (DFS) in ESCC patients of p-SF3B3 (T1200). **(F)** The amino acid sequences surrounding T1200 in SF3B3 in different species are shown. The consensus motif for CK2 phosphorylation is shown at the bottom. **(G)** The specificity of p-SF3B3 (T1200) antibody was examined by using both wild-type (WT, EELDRTPPEVS) and p-T1200 (EELDRT(p)PPEVS) peptides. **(H)** Bacterially expressed, purified His-SF3B3 (WT) and His-SF3B3 (T1200A) proteins were examined by Coomassie blue staining (C.B.S) and indicated by the red arrow. **(I)** In vitro phosphorylation assay was performed by mixing bacterially expressed, purified His-SF3B3 (WT) or His-SF3B3 (T1200A) with or without CK2 kinase, followed by immunoblotting (IB) analysis using antibodies as indicated or Ponceau Staining (P.S). **(J)** KYSE140 cells treated with or without CX-4945 (10 μM, 24 h) were subjected to immunoprecipitation (IP) with anti-SF3B3 antibody, followed by IB analysis with antibodies as indicated. **(K)** HEK293T cells were transfected with GFP-tagged SF3B3 and then treated with or without CX-4945 (10 μM, 24 h), followed by IP analysis with anti-GFP antibody and IB analysis with antibodies as indicated. **(L)** KYSE140 cells were subjected to IP analysis with control IgG or anti-SF3B3 antibody, followed by IB analysis with antibodies as indicated. **(M)** HEK293T cells transfected with FLAG-tagged CSNK2A1 and GFP-tagged SF3B3 were subjected to IP analysis with anti-Flag M2 agarose, followed by IB analysis with antibodies as indicated. **(N)** HEK293T cells transfected with FLAG-tagged SF3B3 and MYC-tagged CSNK2A1 were subjected to IP analysis with anti-Flag M2 agarose, followed by IB analysis with antibodies as indicated. The data underlying the graphs shown can be found in S1 Data.

Protein kinases are known to be dysregulated in a variety of diseases, including cancer [36]. To identify key kinases and druggable targets involved in ESCC, we employed KSEA on large-scale proteomic and phosphoproteomic data from our prior study [1,35]. Our analysis revealed aberrant activity in over 52 protein kinases in ESCC, with 24 kinases exhibiting hyper-activation and 28 kinases showing decreased activity (Fig 1A and S1 Table). Notably, several cell cycle-related kinases, including CDK2, CDK6, CLK1, MAPKs, and ATR, were found to be hyper-activated, consistent with previous studies [1,13,37–40]. Intriguingly, we identified hyper-activation of both catalytic subunits of CK2 kinase, CK2α (CSNK2A1) and CK2α′ (CSNK2A2), in ESCC (Fig 1A). Concurrently, functional enrichment analysis of hyper-phosphorylated proteins revealed a significant enrichment of mRNA splicing-related proteins (Fig 1B and S2 Table). Among these splicing-related proteins, we identified multiple proteins with phosphorylation sites conforming to the CK2 kinase consensus motif (S/TXE/D, S/TXXE/D), including SF3B3 (T1200), SF3B2 (S436), SF3B1 (T299), HNRNPA1L2 (S6), and HNRNPM (S481). Among these, the phosphorylation of SF3B3 at T1200 showed the most significant increase in tumors (Figs 1C and S1A). Furthermore, the levels of T1200 phosphorylation in SF3B3, but not T299 phosphorylation in SF3B1 or S436 phosphorylation in SF3B2, are significantly correlated with prognosis in patients with ESCC (Figs 1D, 1E, and S1B–S1E). Moreover, the amino acid sequence surrounding T1200 is highly conserved across multiple species, underscoring its functional importance (Fig 1F).

To confirm CK2-mediated phosphorylation of SF3B3 at T1200, we generated a custom antibody specifically targeting phosphorylated Thr1200 on SF3B3 (p-SF3B3 (T1200)). Dot blot experiments showed that this antibody specifically recognized peptides containing pT1200, but not wild-type (WT) peptides (Fig 1G). We then expressed and purified recombinant His-tagged, WT SF3B3 and the T1200A mutant in which threonine 1200 was replaced with alanine (Fig 1H). In vitro phosphorylation assay demonstrated that SF3B3 (WT), but not T1200A mutant, could be phosphorylated at T1200 by CK2 in vitro (Fig 1I). Moreover, to assess whether CK2 phosphorylates SF3B3 on T1200 in vivo, we treated KYSE140 cells with CX-4945, a specific CK2 inhibitor, and performed immunoprecipitation using an SF3B3 antibody to examine T1200 phosphorylation levels. The results showed that CK2 inhibition led to a significant reduction in T1200 phosphorylation (Fig 1J). Similar results were observed in 293T cells transfected with GFP-SF3B and subsequently treated with CX-4945 (Fig 1K). It should be noted that there was also a decrease of the total protein levels of SF3B3 (*vida infra*) (Fig 1J and 1K). Next, we investigated whether SF3B3 interacts with CK2. The interaction between SF3B3 and CK2, represented by the catalytic subunit CSNK2A1, was then examined by immunoprecipitation. The results showed that CSNK2A1 was pulled down by SF3B3 (Fig 1L). In addition, the interaction between SF3B3 and CSNK2A1 was confirmed by overexpressed SF3B3 and CSNK2A1 in HEK293T cells (Fig 1M and 1N). These data collectively demonstrate that SF3B3 is a bona fide substrate of CK2, and that CK2-mediated phosphorylation of SF3B3 at T1200 is clinically relevant in ESCC patients.

### SF3B3 functions as an oncoprotein in ESCC both in vitro and in vivo

**Fig 2:**
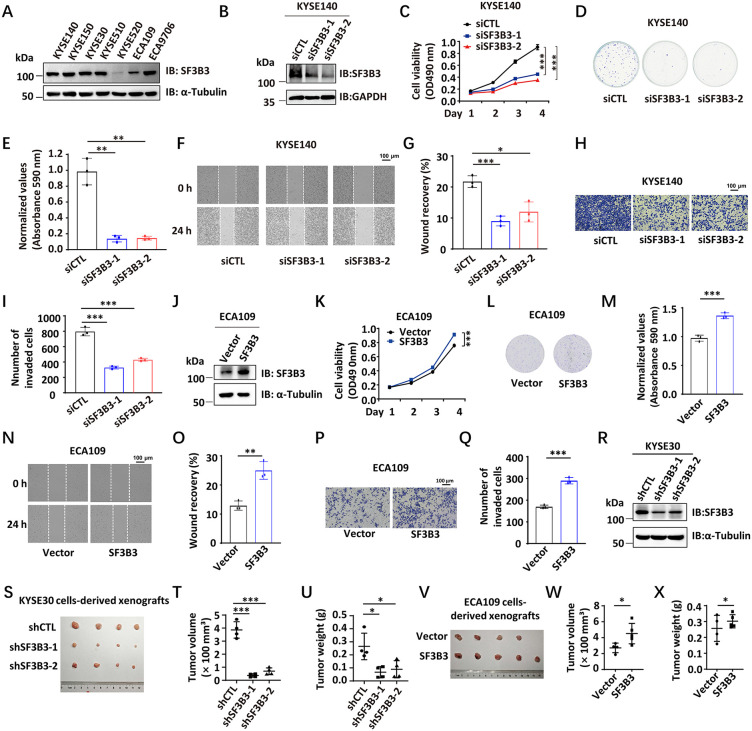
SF3B3 promotes ESCC cell proliferation, colony formation, migration, and invasion in vitro and tumor growth in vivo. **(A)** Seven ESCC cell lines as indicated were subjected to IB analysis to examine SF3B3 protein expression. **(B–D, F, H)** KYSE140 cells were transfected with control siRNA (siCTL) or two individual siRNA targeting SF3B3 (siSF3B3-1 and siSF3B3-2), followed by IB (B), cell proliferation (C), colony formation (D), wound healing (F), and transwell (H) analysis (mean ± SD; ***P < 0.001, Student *t* test). **(E)** The quantification of the crystal violet dye in (D) is shown. (mean ± SD; ***P < 0.001, Student *t* test). **(G)** The quantification of the percentage of wound recovery in (F) is shown. (mean ± SD; *P < 0.05, **P < 0.01, Student *t* test). **(I)** The quantifica*t*ion of the number of invasive cells in (H) is shown. (mean ± SD; **P < 0.01, Student *t* test). **(J–L, N, P)** ECA109 cells stably expressing control len*t*iviral pCDH vector or pCDH-SF3B3 were subjected to IB (J), cell proliferation (K), colony formation (L), wound healing (N), and transwell (P) analysis (mean ± SD; ***P < 0.001, Student *t* test). **(M)** The quantification of the crys*t*al violet dye in (L) is shown (mean ± SD; ***P < 0.001, Student *t* test). **(O)** The quantification of the percentage of wound recovery in (N) is shown (mean ± SD; **P < 0.01, Student *t* test). **(Q)** The quantification of the number of invasive cells in (P) is shown (mean ± SD; ***P < 0.001, Student *t* test). **(R)** KYSE30 cells stably expressing a control shRNA (shCTL) or two independent shRNAs targeting SF3B3 (shSF3B3-1 and shSF3B3-2) were subjec*t*ed to IB analysis. **(S)** KYSE30 cells stably expressing shCTL, shSF3B3-1, or shSF3B3-2 were injected into BALB/c nude mice for 3 weeks, and the tumors were collected and photographed. **(T)** The average of the tumor volume as shown in (S) (mean ± SD; ***P < 0.001, Student *t* test). **(U)** The average of the tumor weight as shown in (S) (mean ± SD; *P < 0.05, Student *t* test). **(V)** ECA109 cells stably expressing control lentiviral pCDH vector or pCDH-SF3B3 were injected into BALB/c nude mice for 2 weeks, and the tumors were collec*t*ed and photographed. **(W)** The average of the tumor volume as shown in (V) (mean ± SD; *P < 0.05, Student *t* test). **(X)** The average of the tumor weight as shown in (V) (mean ± SD; *P < 0.05, Student *t* test). The data underlying *t*he graphs shown can be found in S1 Data.

To further investigate the role of SF3B3 phosphorylation in ESCC progression, we first examined the oncogenic function of SF3B3 in ESCC. The protein expression levels of SF3B3 were assessed in seven different ESCC cell lines (Fig 2A). We then performed SF3B3 knockdown in KYSE140 and KYSE30 cells using two independent siRNAs targeting SF3B3. SF3B3 knockdown significantly inhibited cell proliferation, colony formation, migration, and invasion in both cell lines (Figs 2B–2I and S2A–S2H). Conversely, overexpression of SF3B3 in ECA109 and KYSE520 cells, which have relatively low endogenous SF3B3 expression, promoted cell proliferation, colony formation, migration, and invasion (Figs 2J–2Q and S2I–S2P). To assess the oncogenic role of SF3B3 in vivo, KYSE30 cells stably expressing either a control shRNA (shCTL) or shRNA specifically targeting SF3B3 (shSF3B3) were subcutaneously injected into nude mice. Tumors derived from shSF3B3-expressing cells showed significantly reduced volume and weight compared to those from control cells (Fig 2R–2U). In contrast, overexpression of SF3B3 in ECA109 cells resulted in increased tumor growth in vivo (Fig 2V–2X). Collectively, our findings demonstrate that SF3B3 functions as an oncoprotein in ESCC.

### CK2-mediated phosphorylation of SF3B3 at T1200 is crucial for its oncogenic in ESCC

**Fig 3:**
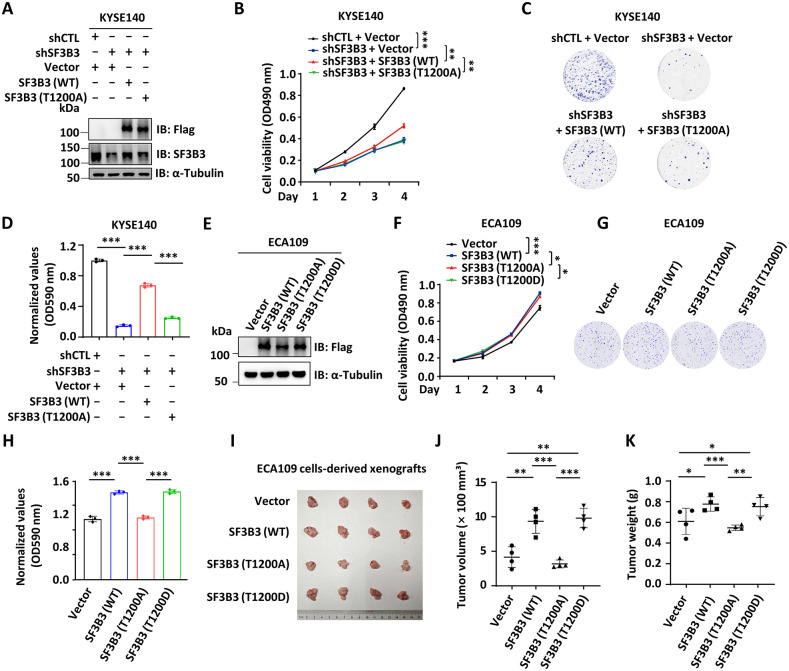
CK2-mediated phosphorylation of SF3B3 at T1200 is crucial for SF3B3 function as an oncoprotein in ESCC. **(A–C)** KYSE140 cells were infected with lentivirus expressing shCTL or shSF3B3 in the presence or absence of lentivirus expressing control vector or wild-type (WT) SF3B3 or T1200A mutant, followed by IB analysis (A), cell proliferation assay (B), and colony formation assay (C) (mean ± SD; *P < 0.05, ***P < 0.001, Student *t* test). **(D)** The quantification of the crystal violet dye in (C) is shown (mean ± SD; *P < 0.05, **P < 0.01, ***P < 0.001, Student *t* test). **(E–G)** ECA109 cells were infected with len*t*ivirus expressing control vector or SF3B3 (WT), SF3B3 (T1200A), or SF3B3 (T1200D), followed by IB analysis (E), cell proliferation assay (F), and colony formation assay (G). **(H)** The quantification of the crystal violet dye in (G) is shown (mean ± SD; *P < 0.05, **P < 0.01, ***P < 0.001, Student *t* test). **(I)** ECA109 cells stably expressing control lentiviral vector, SF3B3 (WT), SF3B3 (T1200A), or SF3B3 (T1200D) were injected in*t*o BALB/c nude mice for 2 weeks, and the tumors were collected and photographed. **(J)** The average of the tumor volume as shown in (I) (mean ± SD; **P < 0.01, ***P < 0.001, Student *t* test). **(K)** The average of the tumor weight as shown in (I) (mean ± SD; *P < 0.05, **P < 0.01, ***P < 0.001, Student *t* test). The data underlying the graphs shown can be found in S1 Data.

To investigate the role of T1200 phosphorylation of SF3B3 in ESCC, we knocked down SF3B3 in KYSE140 cells and reintroduced SF3B3 (WT) or the T1200A mutant at physiologically relevant levels (Fig 3A). As expected, SF3B3 knockdown suppressed cell proliferation in KYSE140 cells. Reintroducing SF3B3 (WT), but not the phosphorylation-defective mutant T1200A, rescued the growth defects caused by SF3B3 knockdown (Fig 3B). Consistently, colony formation was reduced following SF3B3 knockdown and was rescued upon reintroducing of SF3B3 (WT), but not T1200A (Fig 3C and 3D). In line with the observation that SF3B3 protein levels decreased in response to CK2 inhibitor, the expression of SF3B3 (T1200A) was consistently lower than that of SF3B3 (WT) (Fig 3A).

To further explore the critical role of T1200 phosphorylation in the oncogenic function of SF3B3, we established ECA109 cell lines stably expressing a control vector, SF3B3 (WT), the phosphorylation-defective T1200A mutant, and the phosphomimetic mutant (T1200D) mutant (Fig 3E). As expected, stable expression of SF3B3 enhanced cell proliferation and colony formation in ECA109 cells (Fig 3F–3H). Moreover, the volume and weight of tumors derived from ECA109 cells stably expressing SF3B3 were significantly increased compared to those from control cells (Fig 3I–3K). However, the oncogenic potential of SF3B3 was significantly diminished in the T1200A mutant (Fig 3F–3K). Surprisingly, the phosphomimetic mutant (T1200D) did not further enhance the oncogenic potential of SF3B3 (*vida infra*) (Fig 3F–3K). Together, these results demonstrate that CK2-mediated phosphorylation of SF3B3 at T1200 is crucial for its oncogenic in ESCC.

### CK2-mediated phosphorylation of SF3B3 at T1200 stabilizes the SF3B3 protein

**Fig 4:**
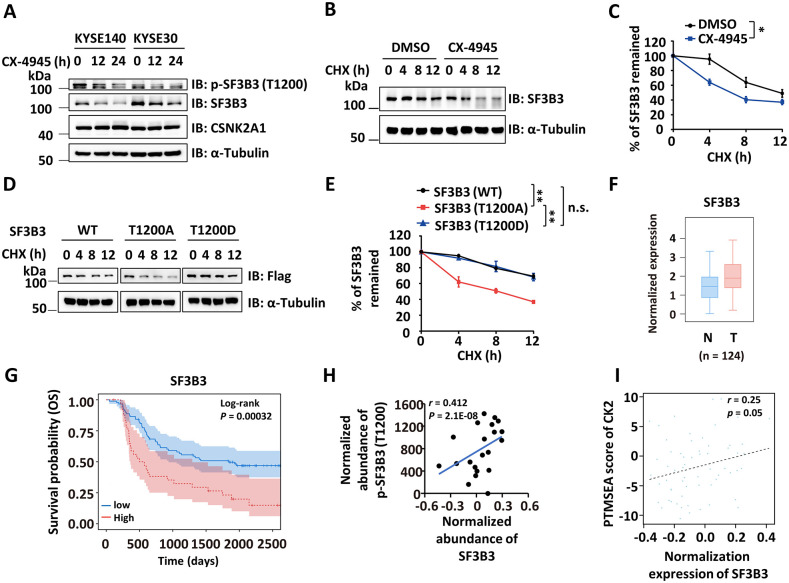
CK2-mediated phosphorylation of SF3B3 at T1200 stabilizes the SF3B3 protein. **(A)** KYSE140 and KYSE30 cells were treated with CX-4945 for 0, 12, and 24 h (10 μM), followed by IB analysis with antibodies as indicated. **(B)** KYSE30 cells were treated with CX-4945 (10 μM) for 12 h and then treated with cycloheximide (CHX) (50 μg/ml) for 0, 4, 8, or 12 h, followed by IB analysis with antibodies as indicated. **(C)** The quantification of SF3B3 proteins in (B) is shown (mean ± SD; *P < 0.05, Student *t* test). **(D)** KYSE30 cells were infected with lentivirus expressing SF3B3 (WT), SF3B3 (T1200A), or SF3B3 (T1200D) for 48 h and then treated with CHX (50 μg/mL) for 0, 4, 8, or 12 hours, followed by IB analysis with antibodies as indicated. **(E)** The quantification of SF3B3 proteins in (D) is shown (mean ± SD; *P < 0.05, ** *P* < 0.01, Student *t* test). **(F)** The rela*t*ive expression of SF3B3 in ESCC tumor (T) and adjacent normal (N) tissues (n = 124) from CPPA database is shown. **(G)** Kaplan–Meier plots of overall survival of SF3B3 in ESCC patients using the CPPA dataset. **(H)** The correlation between the expression of SF3B3 and the abundance of p-SF3B3 (T1200) in ESCC clinical samples as described in Fig 1D is shown. **(I)** The correlation between PTMSEA score of CK2 and the expression of SF3B3 in ESCC clinical samples as described in Fig 1D is shown. The data underlying the graphs shown can be found in S1 Data.

We observed that the SF3B3 protein levels decreased in response to CK2 inhibitor treatment, and the SF3B3 (T1200A) mutant consistently expressed at lower levels than SF3B3 (WT) (Figs 1J, 1K, 3A, and 3E). Given the established link between protein phosphorylation and stability [41,42], we hypothesized that CK2-mediated phosphorylation of SF3B3 at T1200 might regulate its stability. To test this, we first treated KYSE140 and KYSE30 cells with CX-4945 for varying durations. We observed a time-dependent decrease in both T1200 phosphorylation and total SF3B3 protein levels (Fig 4A). We then measured the protein half-life of SF3B3 in KYSE30 cells with or without CX-4945 treatment. CK2 inhibition significantly decreased the half-life of SF3B3 protein (Fig 4B and 4C). Moreover, the phosphorylation-defective mutant T1200A exhibited a significantly shorter half-life. Surprisingly, the half-life of the phosphomimetic mutant T1200D was comparable to that of the WT protein (Fig 4D and 4E). Consistent with the finding for p-SF3B3 (T1200), SF3B3 protein levels were significantly elevated in ESCC tissues compared to adjacent normal tissues, based on analysis from the Cancer Proteome and Phosphoproteome Atlas (CPPA) (Fig 4F) [43]. Furthermore, high expression of SF3B3 was associated with poor prognosis in ESCC patients (Fig 4G). To further support the functional link between CK2 and SF3B3 protein stability, we observed a strong correlation between CK2-mediated SF3B3 phosphorylation at T1200 and SF3B3 protein expression levels, as well as a strong correlation between CK2 kinase activity and SF3B3 protein expression levels in ESCC samples (Fig 4H and 4I). These findings indicate that CK2-mediated phosphorylation of SF3B3 stabilizes the SF3B3 protein.

### Phosphorylation of SF3B3 at T1200 stabilizes the protein via USP7-mediated deubiquitination

**Fig 5:**
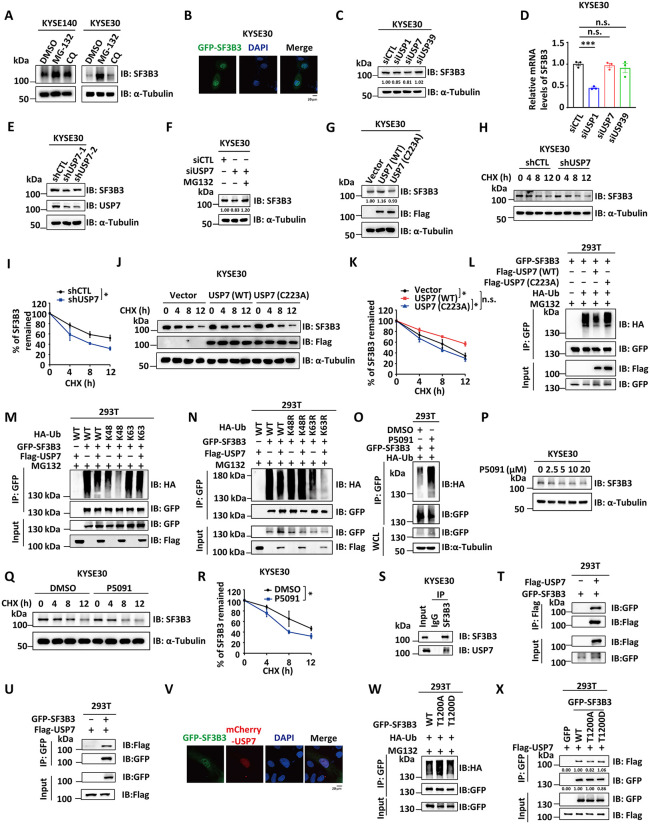
Phosphorylation of SF3B3 at T1200 stabilizes the protein through USP7-mediated deubiquitination. **(A)** KYSE140 and KYSE30 cells were treated with DMSO, MG132 (20 μM), or CQ (20 μM) for 10 h, followed by IB analysis using antibodies as indicated. **(B)** KYSE30 cells were infected with lentivirus expressing GFP-SF3B3, followed by immunofluorescence analysis. GFP-SF3B3 were visualized through GFP (green), and nuclei were indicated by DAPI (blue). Representative images are shown. Scale bars, 20 μm. **(C, D)** KYSE30 cells were transfected with siCTL, siUSP1, siUSP7, or siUSP39 for 48 h, followed by IB (C) and RT-qPCR (D) analysis to assess the protein and mRNA levels of SF3B3, respectively (mean ± SD; ns: not significant, ***P < 0.001, Student *t* test). **(E)** KYSE30 cells stably expressing shCTL or two individual shRNAs targeting USP7 (shUSP7−1 and shUSP7−2) were subjected to IB analysis with antibodies as indicated. **(F)** KYSE30 cells were transfected with siUSP7 and then treated with or without MG132 (20 μM) for 10 h, followed by IB analysis with antibodies as indicated. **(G)** KYSE30 cells stably expressing control lentiviral pCDH vector, USP7 (WT), or USP7 (C223A) were subjected to IB analysis with antibodies as indicated. **(H)** KYSE30 cells stably expressing shCTL or a shRNA targeting USP7 (shUSP7) were treated with CHX (50 μg/ml) for 0, 4, 8, or 12 hours, followed by IB analysis with antibodies as indicated. **(I)** The quantification of SF3B3 proteins in (I) is shown (mean ± SD; *P < 0.05, Student *t* test). **(J)** KYSE30 cells stably expressing control lentiviral pCDH vector, USP7 (WT), or USP7 (C223A) were treated with CHX (50 μg/ml) for 0, 4, 8, or 12 hours, followed by IB analysis with antibodies as indicated. **(K)** The quantification of SF3B3 remained in (K) is shown (mean ± SD; *P < 0.05, Student *t* test). **(L)** HEK293T cells *t*ransfected with GFP-SF3B3 in the presence or absence of HA-Ub, FLAG-USP7 (WT), or USP7 (C223A) were treated with MG132 (20 μM) for 6 h, followed by IP with anti-GFP magnetic beads and IB analysis with antibodies as indicated. **(M)** HEK293T cells transfected with or without GFP-SF3B3 or FLAG-USP7 in the presence or absence of HA-Ub (WT), HA-Ub-K48 (Lys48-only), or HA-Ub-K63 (Lys63-only) were treated with MG132 (20 μM) for 6 h, followed by IP analysis with anti-GFP magnetic beads and IB analysis with antibodies as indicated. **(N)** HEK293T cells transfected with or without GFP-SF3B3 or FLAG-USP7 in the presence or absence of HA-Ub (WT), HA-Ub-K48R, or HA-Ub-K63R were treated with MG132 (20 μM) for 6 h, followed by IP analysis with anti-GFP magnetic beads and IB analysis with antibodies as indicated. **(O)** HEK293T cells were co-transfected with GFP- SF3B3 and HA-Ub for 24 h and treated with or without P5091 (20 μM) for an additional 24 h before treating MG132 (20 μM) for 6 h, followed by IP with anti-GFP magnetic beads and IB analysis with antibodies as indicated. **(P)** KYSE30 cells were treated with P5091 at concentrations as indicated, followed by IB analysis with antibodies as indicated. **(Q)** KYSE30 cells were treated with P5091 (10 μM) for 24 h and then treated with CHX (50 μg/ml) for 0, 4, 8, or 12 h, followed by IB analysis with antibodies as indicated. **(R)** The quantification of SF3B3 proteins in (R) is shown (mean ± SD; *P < 0.05, Student *t* test). **(S)** KYSE30 cells were subjected *t*o IP analysis with control IgG or anti-SF3B3 antibody, followed by IB analysis with antibodies as indicated. **(T)** HEK293T cells transfected with GFP-SF3B3 in the presence or absence of FLAG-USP7 were subjected to IP analysis with anti-Flag M2 agarose and IB analysis with antibodies as indicated. **(U)** HEK293T cells transfected with FLAG-USP7 in the presence or absence of GFP-SF3B3 were subjected to IP with anti-GFP magnetic beads, followed by IB analysis with antibodies as indicated. **(V)** KYSE30 cells were transfected with GFP-SF3B3 and mCherry-USP7 were subjected to immunofluorescence analysis. GFP-SF3B3 was visualized through GFP (green), mCherry-USP7 was visualized through mCherry (red), and nuclei were indicated by DAPI (blue). Representative images are shown. Scale bars, 20 μm. **(W)** HEK293T cells transfected with HA-Ub in the presence of GFP-SF3B3 (WT), SF3B3 (T1200A), or SF3B3 (T1200D) were treated with MG132 (20 μM) for 6 h, followed by IP analysis with anti-GFP magnetic beads and IB analysis with antibodies as indicated. **(X)** HEK293T cells transfected with Flag-tagged USP7 in the presence of GFP-SF3B3 (WT), SF3B3 (T1200A), or SF3B3 (T1200D) were subjected to IP analysis with anti-GFP magnetic beads, followed by IB analysis with antibodies as indicated. The data underlying the graphs shown can be found in S1 Data.

To investigate the mechanism by which SF3B3 phosphorylation stabilizes the protein, we treated KYSE140 and KYSE30 cells with the proteasome inhibitor MG132 and the autophagy inhibitor chloroquine (CQ). The results showed that MG132, but not CQ, significantly increased SF3B3 protein levels, suggesting that SF3B3 is primarily degraded via the ubiquitin-proteasome pathway (Fig 5A). To identify deubiquitinases that regulate SF3B3 stability, we analyzed the subcellular localization of SF3B3 and found that it predominantly resides in the nucleus (Fig 5B). Next, using the CPPA database, we identified nuclear-localized deubiquitinases with a positive correlation to SF3B3 expression [43]. This analysis revealed USP1, USP7, and USP39 as potential candidates (S3A Fig). Among these, only knockdown of USP7 reduced SF3B3 protein levels without affecting its mRNA levels (Figs 5C, 5D, and S3B). The downregulation of SF3B3 by USP7 knockdown was confirmed in KYSE30 cells expressing shRNA targeting USP7 (Fig 5E). To determine whether USP7 regulates SF3B3 degradation through the proteasome pathway, we examined the effect of the proteasome inhibitor MG132 on SF3B3 levels in USP7-depleted ESCC cells. We found that MG132 reversed the decrease in SF3B3 protein levels caused by USP7 depletion (Figs 5F and S3C). Further investigation showed that overexpression of USP7 (WT), but not the catalytically inactive USP7 (C223A) mutant, resulted in increased SF3B3 protein levels (Fig 5G). Cycloheximide chase experiments revealed accelerated degradation of the SF3B3 in USP7-knockdown cells compared to control cells (Fig 5H and 5I). In contrast, delayed degradation was observed in cells overexpressing USP7 (WT), but not in those overexpressing USP7 (C223A) (Fig 5J and 5K).

To examine if USP7 stabilizes SF3B3 by deubiquitination, we analyzed SF3B3 ubiquitination in the presence of the proteasome inhibitor MG132. Overexpression of USP7 (WT), but not the USP7 (C223A) mutant, significantly reduced SF3B3 ubiquitination (Fig 5L). Given that protein degradation is often associated with K48- or K63-linked polyubiquitination and that USP7 preferentially cleaves K48- and K63-linked polyubiquitin chains [44–47]. We found that USP7 reduced the K48-linked, but not K63-linked, polyubiquitination of SF3B3 (Fig 5M and 5N). Furthermore, consistent with USP7 knockdown, treatment with the USP7 inhibitor P5091 significantly increased SF3B3 ubiquitination levels (Fig 5O), decreased SF3B3 protein levels in a concentration-dependent manner (Fig 5P), and reduced SF3B3 protein half-life (Fig 5Q and 5R). To further confirm that USP7 functions as a deubiquitinase for SF3B3, immunoprecipitation experiments demonstrated an interaction between SF3B3 and USP7 in both KYSE140 and KYSE30 cells (Figs 5S and S3D). Co-immunoprecipitation in HEK293T cells with overexpressed SF3B3 and USP7 confirmed this interaction (Fig 5T and 5U). Additionally, co-immunostaining analysis showed co-localization of SF3B3 and USP7 in KYSE30 cells (Fig 5V). Together, these results demonstrate that USP7 interacts with SF3B3 and stabilizes it by cleaving K48-linked polyubiquitin chains.

Prompted by the established crosstalk between protein phosphorylation and ubiquitination [48], we investigated whether T1200 phosphorylation enhances SF3B3 stability through its effect on ubiquitination. We co-transfected HEK293T cells with HA-Ub and GFP-tagged SF3B3 (WT), SF3B3 (T1200A), or SF3B3 (T1200D). Immunoprecipitation assay showed that the T1200A mutant exhibited significantly increased ubiquitination, while the T1200D mutant showed similar ubiquitination levels to SF3B3 (WT) (Fig 5W). This was consistent with the observation that the T1200A mutant is less stable and expressed at lower levels compared to SF3B3 (WT), while the T1200D mutant is similarly stable and expressed comparable levels to SF3B3 (WT) (Figs 3A, 3E, 4D, and 4E).

Given the established link between protein phosphorylation and protein-protein interactions [4,48,49], we hypothesized that phosphorylation of SF3B3 at T1200 might enhance protein stability by promoting its interaction with USP7 and subsequently decreasing its ubiquitination. Co-immunoprecipitation experiments further demonstrated that the T1200A mutant displayed a weaker interaction with USP7 compared to SF3B3 (WT) (Fig 5X). Together, these results collectively showed that phosphorylation of SF3B3 at T1200 enhances its stability through USP7-mediated deubiquitination.

### SF3B3-induced exon inclusion in *EXOSC2* gene is critical for the oncogenic function of SF3B3 in ESCC

**Fig 6:**
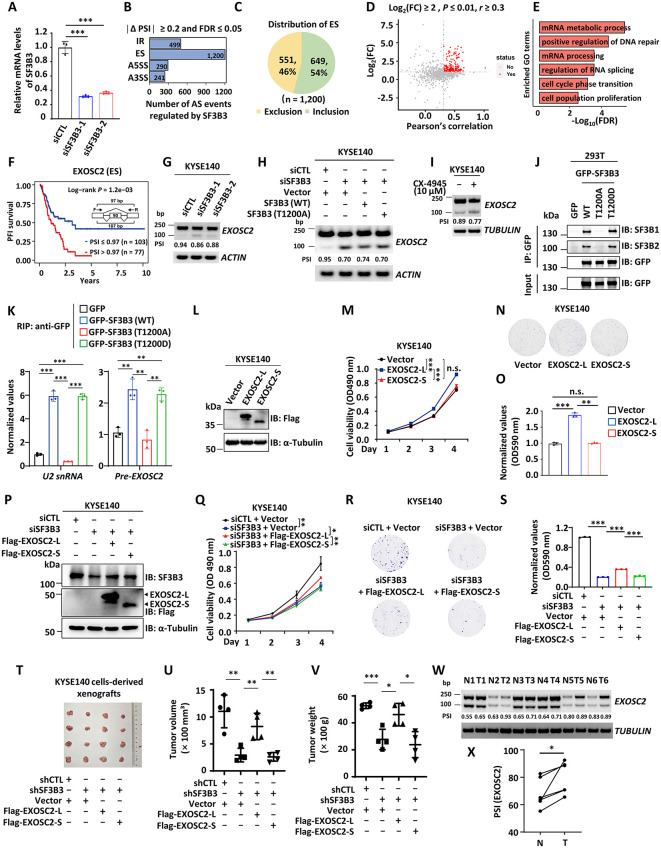
SF3B3-induced exon inclusion in *EXOSC2* gene is critical for the oncogenic function of SF3B3 in ESCC. **(A)** KYSE140 cells transfected with siCTL or two independent siSF3B3 (siSF3B3-1 and siSF3B3-2) were subjected to RT-qPCR analysis to assess mRNA expression levels of SF3B3 (mean ± SD; ***P < 0.001, Student *t* test). **(B)** RNA extracted from cells as described in (A) were subjected to library preparation and Nanopore sequencing, and the number of alternative splicing (AS) events regulated by SF3B3 is shown (|ΔPSI| ≥ 0.2 and FDR ≤ 0.05). Cassette Exons or Exon Skipping (ES); Intron Retain (IR); Alternative 5′ Splice Site (5′ ASS); Alternative 3′ Splice Site (3′ ASS). **(C)** The number of exon inclusion and skipping induced by SF3B3 is shown by pie chart. **(D)** The Pearson’s correlation was analyzed between the expression levels of SF3B3 and genes containing SF3B3-regulated cassette exons that are highly expressed in ESCC (Log_2_FC ≥ 2, *P* ≤ 0.01). Red dots indicate genes with *r* ≥ 0.3. **(E)** GO analysis results for genes with SF3B3-regulated cassette exons. The top 6 most enriched GO terms are shown. **(F)** Kaplan–Meier analyses of progression-free interval in ESCC patients using EXOSC2 cassette exon 4 as an input in the OncoSplicing dataset. **(G)** KYSE140 cells transfected with siCTL, siSF3B3-1, or siSF3B3-2 were subjected to standard PCR analysis to examine the expression of both the short and long isoforms of EXOSC2 as indicated at the bottom. Percentage spliced in (PSI) values were measured by Image J. The position of the cassette exon in EXOSC2 is as follows: *EXOSC2* (NM_001114122, exon 4). DNA marker is indicated on the left. **(H)** KYSE140 cells were transfected with siCTL or siSF3B3 in the presence or absence of control vector, SF3B3 (WT), or SF3B3 (T1200A), followed by standard PCR analysis to examine the short and long isoforms of EXOSC2 as described in (G). **(I)** KYSE140 cells treated with or without CX-4945 (10 μM) were subjected to standard PCR analysis to examine the short and long isoforms of EXOSC2 as described in (G). **(J)** HEK293T cells transfected with GFP control vector or GFP-tagged SF3B3 (WT), SF3B3 (T1200A), or SF3B3 (T1200D) were subjected to IP analysis with anti-GFP magnetic beads, followed by IB analysis with antibodies as indicated. **(K)** HEK293T cells transfected with GFP control vector or GFP-tagged SF3B3 (WT), SF3B3 (T1200A), or SF3B3 (T1200D) were subjected to RIP analysis with anti-GFP magnetic beads, followed by RT-qPCR analysis to examine the binding of SF3B3 protein with genes as indicated (mean ± SD; ***P < 0.001, **P < 0.01, Student *t* test)*.* **(L–N)** KYSE140 cells s*t*ably expressing control lentiviral vector, EXOSC2 long isoform (EXOSC2-L), or EXOSC2 short isoform (EXOSC2-S) were subjected to IB analysis (L), cell proliferation assay (M), and colony formation assay (N) (mean ± SD; ns: not significant, ***P < 0.001, Student *t* test). **(O)** The quantifica*t*ion of the crystal violet dye in (N) is shown (mean ± SD; ns: not significant, **P < 0.01, ***P < 0.001, Student *t* test). **(P–R)** KYSE140 cells transfected with siCTL or siSF3B3, followed by infec*t*ion with lentivirus expressing control vector, EXOSC2-L, or EXOSC2-S were subjected to IB analysis (P), cell proliferation assay (Q), and colony formation assay (R) (mean ± SD; *P < 0.05, **P < 0.01, Student *t* test). **(S)** The quantification of the number of colonies in (R) (mean ± SD; ***P < 0.001, Student *t* test). **(T)** KYSE140 cells infected with lentivirus expressing shCTL or shSF3B3 in the presence or absence of lentivirus expressing control vec*t*or, EXOSC2-L, or EXOSC2-S were injected into BALB/c nude mice for 4 weeks, and the tumors were collected and photographed. (U, **V)** The tumor volume (U) and weight (V) as shown in (T) (mean ± SD; *P < 0.05, **P < 0.01, ***P < 0.001, Student *t* test). **(W)** The expression of the short and long isoforms of EXOSC2 were measured by standard PCR as described in (G) in paired ESCC and adjacent normal tissues (*n* = 6). **(X)** The quantification of the PSI values in (W) (mean ± SD; *P < 0.05, Student *t* test). The data underlying the graphs shown can be found in S1 Data.

SF3B3 is a subunit of the U2 snRNP, involved in recognizing and binding to branch sites within the intron, and plays a key role in regulating AS [28,29]. To investigate SF3B3-regulated AS events and their potential role in ESCC progression, we performed Nanopore sequencing after knocking down SF3B3 in KYSE140 cells (Fig 6A). A total of 2,230 AS events were significantly altered by SF3B3 knockdown, including 1,200 cassette exons (SEs), 499 intron retention (IR), 290 alternative 5′ splice site (5′ ASS), and 241 alternative 3′ splice site (3′ ASS) (|△PSI| ≥ 0.2 and FDR ≤ 0.05) (Fig 6B and S3 Table). Among the SF3B3-regulated cassette exons (*n* = 1,200), 649 were inclusion events, and 551 were exclusion events (Fig 6C and S3 Table).

To identify SF3B3-regulated cassette exons with potential function in ESCC, we analyzed RNA-seq data from The Cancer Genome Atlas (TCGA), focusing on those cassette exons from genes that are highly expressed in ESCC (Log_2_FC ≥ 2, P ≤ 0.01) and positively correlated with SF3B3 expression (*r* ≥ 0.3) (Fig 6D and S4 Table). Gene Ontology (GO) enrichment analysis revealed that these genes were primarily involved in mRNA metabolic processes (Fig 6E and S4 Table). Furthermore, using the OncoSplicing database, we identified SF3B3-induced exon inclusion event (NM_014285, exon 4, Chr9: 130,698,161−130,698,251) in *EXOSC2* as a survival-associated alternative splicing event with high expression predicting poor prognosis in ESCC patients (Fig 6F). RT-PCR analysis confirmed that SF3B3 knockdown increased the short isoform (exon 4 skipped, EXOSC2-S) and decreased the long isoform of EXOSC2 (exon 4 included, EXOSC2-L) in KYSE140 and KYSE30 cells (Figs 6G and S4A). Meanwhile, we knocked down SF3B3 in KYSE140 and KYSE30 cells and performed rescue experiments using either SF3B3 (WT) or SF3B3 (T1200A). The results showed that SF3B3 (WT), but not the T1200A mutant, was able to partially rescue the skipping of exon 4 of *EXOSC2* caused by SF3B3 knockdown (Figs 6H and S4B). Similar results were observed when cells were treated with CX-4945 (Figs 6I and S4C). SF3B3 is primarily known to act as a scaffold protein, which is crucial for maintaining the structural integrity of the SF3b complex and assisting in the assembly of downstream splicing factors [28]. Given the well-established regulatory role of protein phosphorylation in mediating protein-protein interactions and complex assembly [4,48,49], we hypothesized that the phosphorylation of SF3B3 at T1200 specifically promotes its incorporation into the SF3b complex. Co-immunoprecipitation and RNA Immunoprecipitation assays demonstrated that SF3B3 (T1200A) displayed a much weaker interaction with SF3B1/2 and binding with *U2 snRNA* compared to SF3B3 (WT) (Fig 6J and 6K). Consequently, the binding between SF3B3 (T1200A) and its target *EXOSC2* pre-mRNA was much weaker than that of SF3B3 (WT) (Fig 6K).

To investigate the role of EXOSC2-L and EXOSC2-S in ESCC progression, we generated stable cell lines expressing control vector, EXOSC2-L, or EXOSC2-S in KYSE140 and KYSE30 cells (Figs 6L and S4D). Our results showed that overexpression of EXOSC2-L, but not EXOSC2-S, significantly enhanced cell proliferation and colony formation (Figs 6M–6O and S4E–S4G). We next investigated whether EXOSC2-L could rescue the growth defects caused by SF3B3 knockdown. Reintroduction of EXOSC2-L, but not EXOSC2-S, partially reversed the inhibitory effects of SF3B3 knockdown on cell proliferation and colony formation, suggesting that the impact of SF3B3 on cell proliferation and colony formation is mediated, at least in part, through EXOSC2-L (Fig 6P–6S). Furthermore, EXOSC2-L, but not EXOSC2-S, rescued tumor volume and weight in a mouse xenograft model following SF3B3-knockdown in KYSE140 cells (Fig 6T–6V). To further explore the clinical significance of SF3B3-mediated AS of EXOSC2, we examined the expression levels of the two EXOSC2 isoforms in ESCC tumor and adjacent normal tissues (*n* = 6). The results showed that a significant increase in the percentage of EXOSC2-L in tumor compared to adjacent normal tissues (Fig 6W and 6X). Taken together, these findings suggest that SF3B3-induced exon inclusion in *EXOSC2* is critical for SF3B3’s oncogenic function in ESCC.

### The combination treatment with the CK2 inhibitor CX-4945 and the USP7 inhibitor P5091 exhibits synergistic effects in inhibiting ESCC progression

**Fig 7:**
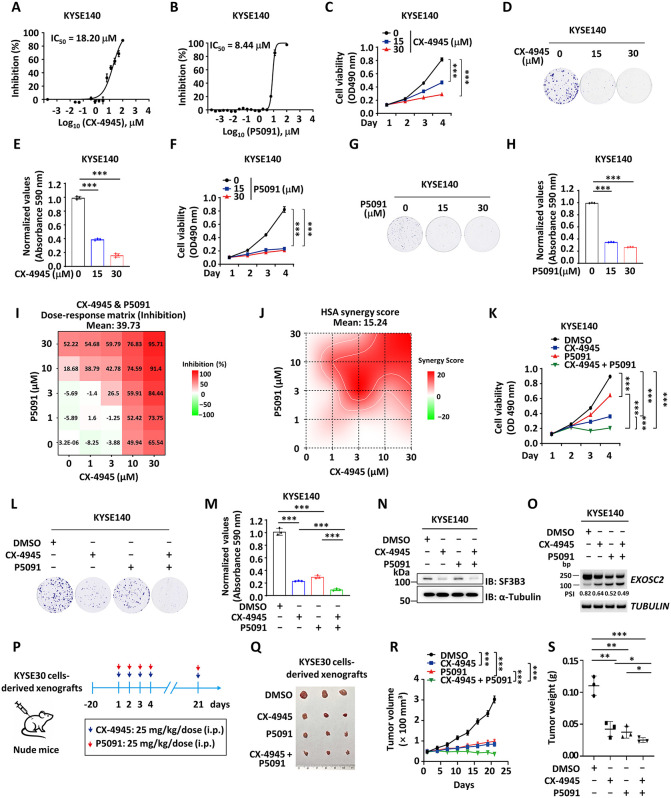
The combination treatment with CX-4945 and P5091 exhibits synergistic effects on inhibiting ESCC progression. **(A, B)** KYSE140 cells were treated with CX-4945 (A) or P5091 (B) at concentrations as indicated for 72 h before cell viability measurement, and the IC50 is shown. (C, D, F, **G)** KYSE140 cells were treated with CX-4945 or P5091 at concentrations as indicated, followed by cell proliferation (C, F) and colony formation (D, G) assay (mean ± SD; ***P < 0.001, Student *t* test). **(E, H)** The quantification of the crystal violet dye in (D) (E) and (G) (H) is shown (mean ± SD; ***P < 0.001, Student *t* test). **(I)** An inhibitory dose-response matrix of CX-4945 and P5091 was conducted in KYSE140 cells. **(J)** Synergy heatmaps representing combination treatment with CX-4945 and P5091 for 3 days in KYSE140. Red color denotes drug synergy. HSA: Highest Single Agent. **(K, L)** KYSE140 cells were treated with CX4945 (10 μM) or P5091 (20 μM) alone or in combination followed by cell proliferation (K) and colony formation (L) assay (mean ± SD, ***P < 0.001, Student *t* test). **(M)** The quan*t*ification of the crystal violet dye in (L) is shown (mean ± SD, ***P < 0.001, Student *t* test). **(N, O)** KYSE140 cells were trea*t*ed with CX4945 (10 μM) or P5091 (20 μM) alone or in combination followed by IB analysis (N) to examine the protein levels of SF3B3 and standard PCR analysis (O) to examine the alternative splicing of EXOSC2. **(P)** Male BALB/c nude mice were subcutaneously injected with KYSE30 cells for 20 days and then randomized (3 mice/group) and treated with CX4945 (25 mg/kg) or P5091 (25 mg/kg) alone or in combination as indicated by intraperitoneal (i.p.) injection daily before tumor collection. **(Q)** Images of excised tumors in (P) are shown. **(R, S)** The tumor growth curve (R) and tumor weight (S) as described in (P) are shown (mean ± SD; *P < 0.05, **P < 0.01, ***P < 0.001, Student *t* test). The data underlying the graphs shown can be found in S1 Data.

Given the oncogenic role of SF3B3 in ESCC and its regulation by both CK2 and USP7, we hypothesized that targeting CK2 and USP7 could effectively inhibit ESCC progression. To test this, we first assessed the cytotoxic effects of CK2 inhibitor CX-4945 and USP7 inhibitor P5091 in ESCC cells. Both inhibitors showed significant cytotoxicity in KYSE140 and KYSE30 cancer cells (Figs 7A, 7B, S5A, and S5C). In contrast, CX-4945 exhibited low toxicity in normal esophageal epithelial Het-1A cells (S5B Fig). Additionally, CX-4945 and P5091 effectively inhibited cell proliferation and colony formation in a dose-dependent manner (Figs 7C–7H and S5C–S5I). Since the CK2-mediated phosphorylation of SF3B3 promotes its deubiquitination and stabilization by USP7, we proposed that combined inhibition of USP7 and CK2 might elicit a synergistic effect on the suppression of ESCC cell proliferation. Using SynergyFinder 2.0, we generated an inhibitory dose-response matrix, which revealed a dose-dependent increase in cytotoxicity with combined treatment (Fig 7I and 7J). The synergy score for the combination of CX-4945 and P5091 is 15.24, suggesting a synergistic interaction between the two compounds (Fig 7J). Subsequently, we investigated the effect of the combination of these two inhibitors on ESCC cell proliferation and colony formation. The combination treatment displayed superior inhibitory effects compared to either drug alone in both KYSE140 and KYSE30 cells (Figs 7K–7M and S5J–S5L). As expected, the combination treatment of CX-4945 and P5091 more effectively reduced SF3B3 protein levels and promoting EXOSC2 exon 4 skipping compared to monotherapy (Figs 7N, 7O, S5M, and S5N). Consistent with our findings in cultured cells, combination treatment with CX-4945 and P5091 exhibited synergistic effects on inhibiting tumor growth in mouse xenograft tumor models using KYSE30 cells, without significant impact on body weight, and the morphology and weight of various organs including liver, spleen, lung, kidney, and heart (Figs 7P–7S and S5O–S5R).

## Discussion

Protein kinase activity is known to be dysregulated in many diseases, including cancer [50,51]. In this study, we found that CK2 is aberrantly activated in ESCC through KSEA analysis of large-scale proteomic and phosphoproteomic data. Among the highly phosphorylated proteins, the splicing factor SF3B3 was notably prominent, as elevated levels of Thr1200 phosphorylation in SF3B3 were significantly correlated with unfavorable patient prognosis. While the SF3B1 subunit of the U2 snRNP is frequently mutated in cancer and its oncogenic role is well-established [30,31,47,52], the function of SF3B3 and its phosphorylation in cancer progression has remained largely unexplored. We demonstrated that CK2 phosphorylates SF3B3 at T1200, a modification crucial for its oncogenic function in ESCC. Mechanistically, CK2-mediated phosphorylation stabilizes SF3B3 by enhancing its interaction with the deubiquitinase USP7. Furthermore, we found that CK2-mediated SF3B3 phosphorylation perturbs the stoichiometric balance of the spliceosomal complex, thereby influencing splicing fidelity and isoform selection. While the significant elevation of SF3B3 T1200 phosphorylation in ESCC tumors (n = 21 paired samples) is a key preliminary finding, the limited sample size necessitates cautious interpretation, and validation in larger, independent cohorts is required to substantiate its potential as a biomarker and therapeutic target.

To investigate the molecular mechanisms underlying the oncogenic role of SF3B3, we identified SF3B3-regulated AS events using long-read sequencing technology. Most of these AS events were cassette exons, with SF3B3-regulated cassette exons significantly enriched in genes involved in mRNA metabolism. Among the numerous AS events regulated by SF3B3, we focused on a clinically relevant event in EXOSC2 (exon 4 inclusion), as EXOSC2 is highly expressed and its expression correlates positively with SF3B3 in ESCC clinical samples. More importantly, exon 4 inclusion in EXOSC2 is associated with prognosis in ESCC patients. EXOSC2, a core component of the RNA exosome, directly participates in mRNA metabolism, but the functional roles of its alternatively spliced isoforms remain unclear [53,54]. Here, we show that the EXOSC2-S isoform, with exon 4 skipped, is downregulated, while the EXOSC2-L isoform is upregulated in ESCC cell lines and clinical samples, indicating its crucial role in ESCC progression. Notably, EXOSC2-L did not fully rescue the growth defects following SF3B3 knockdown, suggesting that SF3B3’s effects on cell growth may involve other genes. Thus, we conclude that SF3B3 partially promotes the malignant phenotypes of ESCC by regulating EXOSC2 AS. However, it will be required to systematically evaluate the association between EXOSC2-L expression and the clinical stage of ESCC. Given that SF3B3 is highly expressed in various cancers, differential inclusion of EXOSC2 exon 4 may be a common event in many human cancers, with EXOSC2 splice variants potentially serving as novel cancer markers. However, the molecular mechanism by which EXOSC2-L promotes cell growth remains to be further investigated.

Although the pharmacological inhibitors CX-4945 and P5091 were used to induce the degradation of SF3B3 and suppress ESCC progression, their broad substrate specificity and potential off-target effects suggest that the observed phenotypes may result from the modulation of multiple downstream pathways rather than exclusively from SF3B3 perturbation. Given the current absence of reported SF3B3-specific inhibitors, future studies will explore targeted degradation and post-translational modification strategies to interrogate SF3B3 function. Specifically, PROTACs (proteolysis-targeting chimeras) may be employed to selectively degrade SF3B3, while PhosTACs (phosphorylation-targeting chimeras) could be utilized to specifically reverse its phosphorylation. These approaches will provide direct mechanistic evidence for the role of SF3B3 in ESCC progression and may uncover novel therapeutic vulnerabilities.

**Fig 8:**
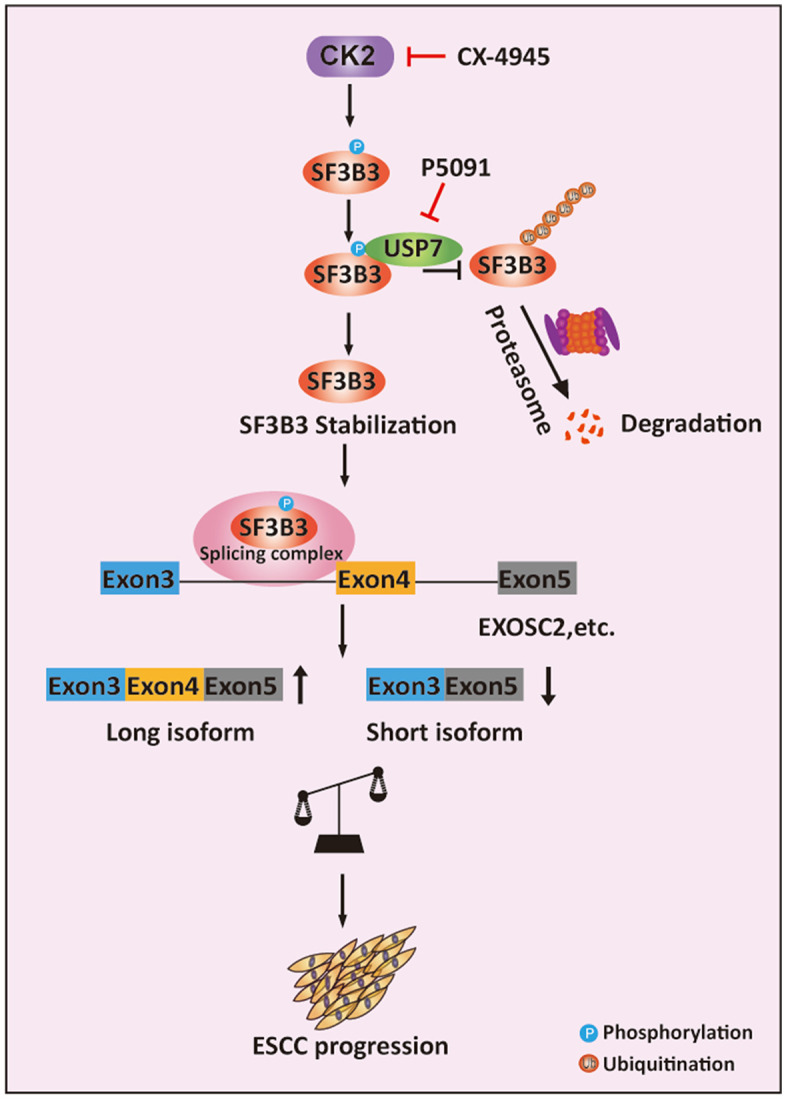
A working model of CK2-mediated SF3B3 phosphorylation in promoting ESCC progression. In ESCC, aberrantly activated CK2 phosphorylates SF3B3 at Thr1200, enhancing its interaction with the deubiquitinase USP7. This interaction leads to deubiquitination and stabilization of SF3B3, ultimately promoting a large cohort of alternative splicing events, including the inclusion of exon 4 in EXOSC2, and thereby driving ESCC progression. Targeting both CK2 and USP7, either individually or in combination, using CX-4945 and P5091, respectively, effectively suppresses ESCC progression.

Based on our findings, we propose a working model in which hyper-activation of CK2 leads to the hyper-phosphorylation of SF3B3 at T1200. This modification enhances the interaction between SF3B3 and USP7, triggering USP7-mediated deubiquitination and stabilization of SF3B3 in ESCC cells. The phosphorylation of SF3B3 at T1200 also facilitates its incorporation into the U2 snRNP complex. The resulting accumulated SF3B3 then drives a wide array of AS events, including the inclusion of exon 4 in EXOSC2 (EXOSC2-L), thereby contributing to tumorigenesis. Importantly, inhibition of CK2 and USP7, either individually or in combination, results in the destabilization of SF3B3 and a balanced expression of EXOSC2-L and EXOSC2-S, resulting in tumor suppression (Fig 8).

## Materials and methods

### Kinase-substrate enrichment analysis (KSEA)

KSEA was performed using the R package of KSEA App to identify potential kinases associated with differential phosphorylation events. This method utilizes kinase-substrate databases, PhosphoSitePlus, and NetworKIN to assign known kinase-substrate relationships to phosphorylation site detected in the phosphoproteomic data we reported previously [1,35,55,56]. Firstly, phosphorylation sites were mapped to their corresponding kinases based on prior knowledge. Next, the normalized enrichment score was calculated for each kinase by comparing the observed changes in the phosphorylation levels of its substrates to the background distribution of all phosphorylation changes. A statistical significance threshold, typically *P* < 0.05, was applied to identify significantly enriched or depleted kinases. The resulting analysis highlights key kinases that may regulate phosphorylation-driven signaling pathways.

### In vitro phosphorylation assay

Bacterially expressed proteins were incubated with CK2 kinase following the manufacturer’s instructions (New England Biolabs, P6010). Briefly, 5 μg protein was incubated with 1× NEBuffer supplemented with 200 μM ATP, 2 μM Na_3_VO_4_, 1 μM NaF, and 500 units of CK2 kinase at 30 °C for 1 h. The reaction was stopped by adding 5 × loading buffer and boiling at 95 °C for 10 min.

### Cell culture

The human ESCC cell lines (KYSE140, KYSE150, KYSE30, KYSE510, KYSE520, ECA109, and ECA9706), and HEK293T cells were used in this study. ECA109 and HEK293T cells were cultured in Dulbecco’s modified Eagle’s medium (DMEM; 01-052-1ACS, Biological Industries) supplemented with 10% fetal bovine serum (FBS; BC-SE-FBS07, Bio-Channel) and 1% penicillin/streptomycin mixture (PS; S110JV, BasalMedia). KYSE140, KYSE510, KYSE520, and KYSE30 cells were cultured in RPMI 1640 medium (01-100-1ACS, Biological Industries) supplemented with 10% FBS and 1% PS mixture. All cells were cultured in a humidified incubator (Thermo Fisher Scientific) at 37 °C with a CO_2_ concentration of 5%.

### Antibodies and other reagents

Rabbit anti-SF3B3 antibody (14577-1-AP), rabbit anti-CSNK2A1 antibody (10992-1-AP), and mouse anti-β-Actin antibodies were purchased from Proteintech. Rabbit anti-GFP antibody (AE078), mouse anti-α-Tubulin antibody (AC012), and magnetic beads-conjugated anti-GFP VHH single domain antibody (AE079) were purchased from ABclonal. Mouse anti-Flag antibody (F1804) and anti-Flag M2 affinity gel (A2220) were purchased from Sigma-Aldrich. Mouse anti-Myc antibody (9E10) was purchased from Santa Cruz Biotechnology. Rabbit IgG2a (BP0089) was purchased from BioXCell. Rabbit anti-Phospho-SF3B3 (Thr1200) antibody was commercially made by ABclonal (antigen peptide: EELDRT(p)PPEVS). Chloroquine (HY-17589A) and DAPI dihydrochloride (HY-D0814) were purchased from MedChemExpress. The proteasome inhibitor MG-132 (T2154), the CK2 inhibitor CX-4945 (T2259), and the USP7 inhibitor P5091 (T6925) were purchased from TargetMol.

### Cell proliferation assay

Cell viability was measured by using a CellTiter 96 AQueous one solution cell proliferation assay kit (G3582, Promega) following the manufacturer’s protocol. Briefly, cells were either transfected with siRNAs or treated with CX-4945 and/or P5091, and then maintained in culture medium for different time followed by cell proliferation assay. To measure cell viability, 20 μL of CellTiter 96 AQueous one solution reagent was added per 100 μL of culture medium, and the culture plates were incubated for 1 hour (h) at 37 °C in a humidified, 5% CO_2_ atmosphere incubator. Data were recorded at a wavelength of 490 nm using a Spark spectrophotometer (Tecan) Microplate Reader.

### Colony formation assay

Cells were seeded into 6-well plates (800 cells per well) and growth media were replenished every three days. The relative cell number was determined by crystal violet staining. Briefly, cells were fixed with a methanol/acetic acid solution (3:1) for five minutes, followed by staining with 0.1% crystal violet (Sango Biotech, A100528-0025) for 15 min (min). To quantify cell numbers, the crystal violet dye was extracted into 10% acetic acid and its absorbance was measured at a wavelength of 590 nm (OD590).

### Wound healing and transwell assay

For wound healing assays, ESCC cells transfected with siRNAs were re-seeded into 6-well plates to form a confluent monolayer. A linear wound was made by scratching with a 200 μL pipette tip and cells were washed three times with phosphate-buffered saline (PBS). Cells were then incubated in fresh media with mitomycin at 5 μg/ml to block cell proliferation. Images of wound recovery were captured every 12 h using a phase contrast microscope (Carl Zeiss). For transwell assay, cell invasion was investigated by a transwell chamber containing 50 ng/ml Matrigel (Corning) according to the manufacturer’s instructions. Briefly, siRNA-transfected cells were re-suspended in 200 μL serum-free medium and loaded onto the upper chamber at a density of 5 × 10^4^ cells per chamber. Meanwhile, 600 μL of culture medium supplemented with 10% FBS was carefully added to the lower chamber. After incubation for 24 h, the inserts were washed with PBS and fixed with 4% paraformaldehyde for 15 min and stained with 0.1% crystal violet for 15 min. Following extensive washing with PBS, non-invaded cells remaining in the upper compartment were removed using a cotton swab. Subsequently, invaded cells were photographed and quantified using Image J software.

### Xenograft tumor assays

Male BALB/c nude mice (4–5 weeks of age, 18–20 g of weight) were used for xenograft tumor models and maintained under SPF conditions in accordance with a protocol approved by the Animal Ethics Committee of Xiamen University (XMULAC20210126). For xenograft models, KYSE30 cells (3 × 10^6^ in 100 μL sterile PBS) transfected with shCTL or shSF3B3, or ECA109 cells (3 × 10^6^ in 100 μL sterile PBS) transfected with control vector or pCDH-SF3B3 were subcutaneously injected in the right flanks of the mice. All mice were euthanized before the tumor burden exceeded the limit (⁓1,500 mm^3^). Tumors were then excised, photographed, and weighted. To examine the effects of combination treatment with CX-4945 and P5091, KYSE30 cells (3 × 10^6^ in 100 μL sterile PBS) were subcutaneously injected in the right flanks of the mice. Mice were randomized into four groups (*n* = 5 in each group) when the tumor size reached approximately 50 mm^3^, mice were treated with vehicle, CX-4945 (25 mg/kg), P5091 (25 mg/kg), or in combination by intraperitoneal injection daily. Tumors volume and mice weight were measured every third day. The tumor volume was calculated using the formula: *V* = 1/2 (length × width^2^). Then, mice were euthanized and subcutaneous tumor tissues were dissected, weighed, and photographed at the end of the experiments.

### SiRNA transfection, RNA isolation, RT-qPCR, and standard PCR

SiRNA (targeting sequences: 5′-CCAAGAAACTCGAGGATAT-3′ (siSF3B3-1); 5′-CCACGAAAGCTCAGAGAAA-3′ (siSF3B3-2); USP1: 5′- GCATAGAGATGGACAGTAT-3′ (siUSP1); USP7: 5′-GCATAGTGATAAACCTGTA-3′ (siUSP7); USP39: 5′-CCATGAGGATCTTCACTAA) transfections were performed using Lipofectamine 2000 according to the manufacturer’s protocol. Total RNA was isolated using RNAiso Plus (Takara), and first-strand cDNA synthesis was performed using HiScript II Q RT SuperMix for qPCR (Vazyme) according to the manufacturer’s protocol. Quantitative PCR (qPCR) was performed using AriaMx Real-Time PCR machine (Agilent Technologies). All RT-qPCRs were repeated at least three times, and the relative abundance of each transcript was normalized to the expression level of ACTIN. Standard PCR was used to examine AS change using 2 × Rapid Taq Plus Master Mix (Vazyme). PCR products were separated on 2% agarose gels, and the amount of each splicing isoform was measured with ImageJ. Sequence information for all the primers used were presented in S5 Table.

### Plasmids transfection, lenti-viral vectors packaging, and infection

Plasmids transfection was performed using Lipofectamine 2000 (Life Technology) or Polyethyleneimine (PEI, Polysciences) according to the manufacturer’s protocol. Lentiviral vector packaging and infection was described previously [57]. Briefly, HEK293T cells were seeded in culture plates coated with poly-D-lysine (0.1% (w/v), Sigma, P7280) and transfected with lentiviral vector together with packaging vectors, psPAX2 (12260, Addgene) and pMD2.G (12259, Addgene), at a ratio of 2:1:1 using PEI. After 48 h, virus was collected, filtered, and added to ESCC cells in the presence of 10 μg/mL polybrene (Sigma, H9268), followed by centrifugation for 30 min at 1,500 *g* at 37 °C. Medium was replaced 24 h later.

### Cloning procedures

SF3B3, EXOSC2, and USP7 were PCR-amplified from complementary DNA (cDNA) derived from HEK293 cells by using Phanta Max Super-Fidelity DNA Polymerase (Vazyme, P505-d1) and then cloned into pCDH-3 × Flag- 3 × HA-EF1-puro (System Biosciences), pCDH-mCherry-puro (System Biosciences), pEGFP-C2 (Addgene), or pET-28a (Novagen) expression vector. The following mutations were generated by overlap extension PCR method using Phanta Max Super-Fidelity DNA Polymerase: SF3B3 phosphorylation-deficient mutant SF3B3 T1200A, SF3B3 phosphorylation mimic mutation SF3B3 T1200D, and USP7 enzymatically dead mutant USP7 C223A. Short hairpin RNAs (shRNAs) targeting SF3B3 (shSF3B3#1, CCAAGAAACTCGAGGATATCC; shSF3B3#2, AGACAGATGAAGATATGGTTA) or USP7 (shUSP7#1, CCTGGATTTGTGGTTACGTTA; shUSP7#2, CAGCTAAGTATCAAAGGAAA) were cloned into the lentiviral vector pLKO.1 between AgeI and EcoRI sites.

### Immunoblotting and immunoprecipitation

Cells were lysed in lysis buffer (50 mM Tris-HCl (pH 7.4), 150 mM NaCl, 1 mM EDTA, and 1% Triton X-100) containing protease inhibitor cocktail (Sigma, P2714-1BTL) and phosphatase inhibitor cocktail (Sigma, 4906837001) on ice for 30 min followed by centrifugation. For immunoblotting, the resultant supernatant was directly boiled in SDS sample buffer, resolved by SDS-PAGE gel. For immunoprecipitation, the resultant supernatant was incubated with antibodies (2–5 μg) at 4 °C overnight. Protein A/G Magnetic Beads (MCE, HY-K0202) were then added and incubated for an additional 4 h before washing five times with washing buffer (the same as lysis buffer). For immunoprecipitation, FLAG- and GFP-tagged proteins were immunoprecipitated with anti-FLAG M2 Affinity Gel (Sigma, A2220) and anti-GFP (Nanobody) Magnetic Beads (ABclonal, AE079), respectively, according to the manufacturer’s protocol. Flag-tagged proteins were then eluted by 3 × FLAG peptides (MDYKDHDGDYKDHDIDYKDDDDK) and boiled in SDS sample buffer, and GFP-tagged proteins were directly boiled in SDS sample buffer The associated proteins were resolved by SDS-PAGE gel.

### RNA Immunoprecipitation

Cells were lysed in lysis buffer (150 mM KCl, 25 mM Tris-HCl (pH 7.4), 0.5 mM dithiothreitol (DTT), 0.5% NP-40) supplemented with protease inhibitor cocktail (sigma, P2714-1BTL), phosphatase inhibitor cocktail (Sigma, 4906837001), and RNase inhibitors (Thermo Fisher Scientific, 10777019), followed by centrifugation. Subsequently, the supernatant was incubated with anti-GFP (Nanobody) Magnetic Beads (ABclonal, AE079) at 4 °C for 4 h. After washing with lysis buffer for 5 times, the bead-bound immunocomplexes were eluted using elution buffer (50 mM Tris-HCl (pH 8.0), 1% SDS, and 10 mM EDTA) at 55 °C for 20 min. To isolate protein-associated RNAs from the eluted immunocomplexes, samples were treated with proteinase K (Beyotime, ST532) at a final concentration of 1 mg/mL, and RNAs were extracted by RNAiso Plus (TaKaRa, 9109) before RT-PCR analysis.

### Protein purification

His-tagged proteins were expressed in BL21 (DE3) bacterial cells (Stratagene) and purified by using Ni-NTA Resin (Thermo Fisher Scientific) according to the manufacturer’s protocol.

### Immunofluorescence

Cells were seeded on coverslips coated with poly-D-lysine in 6-well plates followed by transfection as described above. After 48 h, cells were washed twice with PBS and fixed with 4% paraformaldehyde at room temperature for 20 min. Cells were then washed again with PBS and incubated with 0.1% (v/v) Triton X-100 (Sigma) in PBS for 10 min at RT. Coverslips were mounted onto slides using Mowiol, and DAPI was added for nuclear visualization. Imaging was performed with a Carl Zeiss laser confocal microscope.

### Clinical tissue specimens

Tumor specimens and adjacent normal esophageal epithelial tissues were collected from patients with ESCC who underwent esophagectomy at the Department of Thoracic Surgery, Fujian Medical University Union Hospital, between 2015 and 2018. All patients were treatment-naïve, having received neither radiotherapy nor chemotherapy prior to surgical intervention. Immediately following resection, tissue samples were flash-frozen in liquid nitrogen and subsequently stored at −80 °C until RNA or protein extraction. Histopathological diagnoses were independently confirmed by two experienced pathologists in accordance with the eighth edition of the American Joint Committee on Cancer (AJCC) and the Union for International Cancer Control (UICC) staging criteria. This study was conducted with the approval of the Ethics Committee of Fujian Medical University Union Hospital, and written informed consent was obtained from all participants prior to tissue collection and analysis.

### Nanopore sequencing

Total RNA (50 ng) was reverse transcribed into cDNA using Maxima H Minus Reverse Transcriptase (Thermo Fisher Scientific) according to the manufacturer’s instructions. To amplify the cDNA, PCR reactions were performed using the PCR-cDNA Barcoding kit (SQK-PCB109) protocol. Subsequently, PCR products were treated with exonuclease I (NEB) at 37 °C for 30 min to remove excess primers. After purification with Agencourt AMPure XP magnetic beads (Beckman Coulter), libraries were constructed using the Ligation Sequencing Kit (SQK-LSK109) (Oxford Nanopore) and sequenced with SpotON FlowCell MKⅠ (R9.4) (Oxford Nanopore) according to the manufacture’s protocol. Basecalling from raw data (FAST5 format) was performed using the Guppy software (version 3.0.3) (Oxford Nanopore) [58]. All RNA-seq data were deposited in the Gene Expression Omnibus database under accession GSE285651. The following link has been created to allow review of record GSE285651 while it remains in private status (https://www.ncbi.nlm.nih.gov/geo/query/acc.cgi?acc=GSE285651).

### Alternative splicing (AS) analysis

AS analysis was conducted using FLAIR (Full-Length Alternative Isoform RNA), a tool specifically designed for long-read sequencing data [59]. The pipeline begins by aligning long-read RNA sequencing data to the reference genome using minimap2 [60]. FLAIR identifies full-length isoforms by clustering aligned reads and assigns them to specific splicing events, such as exon skipping, alternative 5′ or 3′ splice sites and intron retention. Differential splicing analysis was subsequently performed by quantifying isoform expression across samples and identifying splicing events with significant differences, using |ΔPSI| ≥ 0.2.

### Quantification and statistical analysis

ImageJ software was used for quantification of WB and RT-PCR data. All statistical analyses were performed using GraphPad Prism 8 software (version 8). For comparisons between two groups, a two-tailed Student *t* test or paired *t* test was applied. Multiple comparisons were analyzed using two-way ANOVA. Survival curves were generated using the Kaplan–Meier method and compared with the log-rank (Mantel-Cox) test. *P* < 0.05 was considered statistically significant.

## Supporting information

S1 Fig CK2-mediated SF3B1/2/3 phosphorylation and its clinical relevance in ESCC patients. **(A)** Tandem MS spectrum of T1200 phosphorylation detected in SF3B3 is shown. **(B, D)** The abundance of p-SF3B1 (T299) (B) and p-SF3B2 (S436) (D) in ESCC tumor (T) and adjacent normal (N) tissues is shown (*n* = 21). **(C, E)** Kaplan–Meier plots of DFS in ESCC patients of p-SF3B1 (T299) (C) and p-SF3B2 (S436) (E). The data underlying the graphs shown can be found in S1 Data.
(TIF)

S2 Fig SF3B3 promotes ESCC cell proliferation, colony formation, migration, and invasion in vitro and tumor growth in vivo. **(A–C, E, G)** KYSE30 cells were transfected with siCTL, siSF3B3-1, or siSF3B3-2, followed by IB analysis (A), cell proliferation assay (B), colony formation assay (C), wound healing assay (E), and transwell assay (G) (mean ± SD; ***P < 0.001, Student *t* test). **(D)** The quantification of the crystal violet dye in (C) is shown (mean ± SD; **P < 0.01, Student *t* test). **(F)** The quantification of the percentage of wound recovery in (E) is shown (mean ± SD; *P < 0.05, ***P < 0.001, Student *t* test). **(H)** The quantifica*t*ion of the number of invasive cells in (G) is shown (mean ± SD; ***P < 0.001, Student *t* test). **(I–K, M, O)** KYSE520 cells were infected with len*t*ivirus expressing control vector or SF3B3 were subjected to IB analysis (I), cell proliferation assay (J), colony formation assay (K), wound healing assay (M), and transwell assay (O) (mean ± SD; **P < 0.01, Student *t* test). **(L)** The quantification of the crys*t*al violet dye in (K) is shown (mean ± SD; ***P < 0.001, Student *t* test). **(N)** The quantification of the percentage of wound recovery in (M) is shown (mean ± SD; **P < 0.01, Student *t* test). **(P)** The quantification of the number of invasive cells in (O) is shown (mean ± SD; **P < 0.01, Student *t* test). The data underlying the graphs shown can be found in S1 Data.
(TIF)

S3 Fig CK2-mediated phosphorylation of SF3B3 at T1200 is crucial for SF3B3 function as an oncoprotein in ESCC. **(A)** The Pearson’s correlation of the expression levels between SF3B3 and USP1, USP7, or USP39 in ESCC in CPPA are shown. **(B)** KYSE30 cells transfected with siCTL, siUSP1, siUSP7, or siUSP39 for 48 h were subjected to RT-qPCR analysis to examine the mRNA levels of USP1, USP7, and USP39 (mean ± SD; *P < 0.05, **P < 0.01, ***P < 0.001, Student *t* test). **(C)** KYSE140 cells were *t*ransfected with siCTL or siUSP7 and then treated with or without the proteasome inhibitor MG132 (20 μM) for 10 h before IB analysis with antibodies as indicated. **(D)** KYSE140 cells were subjected to IP with control IgG or anti-SF3B3 antibody, followed by IB analysis with antibodies as indicated. The data underlying the graphs shown can be found in S1 Data.
(TIF)

S4 Fig SF3B3-induced exon inclusion in *EXOSC2* gene is critical for the oncogenic function of SF3B3 in ESCC. **(A)** KYSE30 cells transfected with siCTL, siSF3B3-1, or siSF3B3-2 were subjected to RT-PCR analysis to examine the expression of both short and long isoforms of EXOSC2 as indicated. PSI values were measured by Image J. **(B)** KYSE30 cells were transfected with siCTL or siSF3B3 in the presence or absence of control vector, SF3B3 (WT), or SF3B3 (T1200A), followed by standard PCR analysis. **(C)** KYSE30 cells treated with or without CX-4945 (10 μM) were subjected to standard PCR analysis to examine the alternative splicing of EXOSC2. **(D–F)** KYSE30 cells stably expressing control pCDH vector, EXOSC2-L, or EXOSC2-S were subjected to IB analysis (D), cell proliferation assay (E), and colony formation assay (F). **(G)** The quantification of the crystal violet dye in (F) is shown (mean ± SD; ns: not significant, **P < 0.01, ***P < 0.001, Student *t* test). The data underlying the graphs shown can be found in S1 Data.
(TIF)

S5 Fig Combination treatment with CX-4945 and P5091 exhibits synergistic effects on inhibiting ESCC progression. **(A–C)** KYSE30 and Het-1A cells were treated with CX-4945 (A, B) or P5091 (C) at concentrations as indicated for 72 h before cell viability measurement, and the IC50 are shown. **(D, E)** KYSE30 cells were treated with CX-4945 at concentrations as indicated, followed by cell proliferation (D) and colony formation (E) assay. **(F)** The quantification of the crystal violet dye in (E) is shown (mean ± SD; ***P < 0.001, Student *t* test). **(G, H)** KYSE30 cells were treated with P5091 at concentrations as indicated, followed by cell proliferation (G) and colony formation (H) assay. **(I)** The quantification of the crystal violet dye in (H) is shown (mean ± SD; ***P < 0.001, Student *t* test). **(J, K)** KYSE30 cells were treated with CX4945 (10 μM) and P5091 (10 μM) alone or in combination, followed by cell proliferation (J) and colony formation (K) assay. **(L)** The quantification of the crystal violet dye in (K) is shown (mean ± SD; ***P < 0.001, Student *t* test). **(M, N)** KYSE30 cells were *t*reated with CX4945 (10 μM) and P5091 (20 μM) alone or in combination, followed by IB analysis (M) using antibodies as indicated and RT-PCR analysis (N) to examine the alternative splicing of EXOSC2. **(O)** The body weight of mice as described in Fig 7P is shown. **(P)** The organs as indicated from mice shown in Fig 7P are shown. **(Q)** The weight of the organs as described in (P) are shown. **(R)** The sections from organs as shown in (P) were subjected to hematoxylin and eosin (H&E) staining, and representative images are shown. The data underlying the graphs shown can be found in S1 Data.
(TIF)

S1 Table The list of dysregulated kinases predicted by KSEA analysis. (XLSX)

S2 Table KEGG pathway, Reactome Gene Sets, and Hallmark Gene Sets enrichment analysis for differential phosphoproteins in ESCC. (XLSX)

S3 Table The list of SF3B3-regulated AS events identified using Nanopore sequencing in KYSE140 cells. (XLSX)

S4 Table The list of genes containing SF3B3-regulated cassette exons that are highly expressed in ESCC (Log_2_FC ≥ 2, *P* ≤ 0.01) and show positive correlation with SF3B3 expression (*r* ≥ 0.3). (XLSX)

S5 Table Sequence information for qPCR and RT-PCR primers used in the current study. (XLSX)

S1 Data Underlying data for Figs 1–7 and S1– S5.
(XLSX)

S1 Raw Images The original, uncropped images for all blots and electrophoresis gels presented throughout the manuscript. (PDF)

## Abbreviations

AS: alternative splicing
CPPA: Cancer Proteome and Phosphoproteome Atlas
CQ: chloroquine
CSNK2A1: casein kinase II subunit α
DUB: deubiquitinating enzyme
ESCC: esophageal squamous cell carcinoma
FBS: fetal bovine serum
GO: Gene Ontology
KSEA: kinase-substrate enrichment analysis
PBS: phosphate-buffered saline
qPCR: quantitative PCR
TCGA: The Cancer Genome Atlas
U2 snRNP: U2 small nuclear ribonucleoprotein
WT: wild-type
