# Supplementary figures and images for: Casein kinase 2-mediated phosphorylation of the splicing factor SF3B3 plays a key role in esophageal squamous cell carcinoma progression
Source: PLoS Biol. 2026 Apr 10;24(4):e3003729. doi: 10.1371/journal.pbio.3003729 (PMC13068320; doi:10.1371/journal.pbio.3003729)

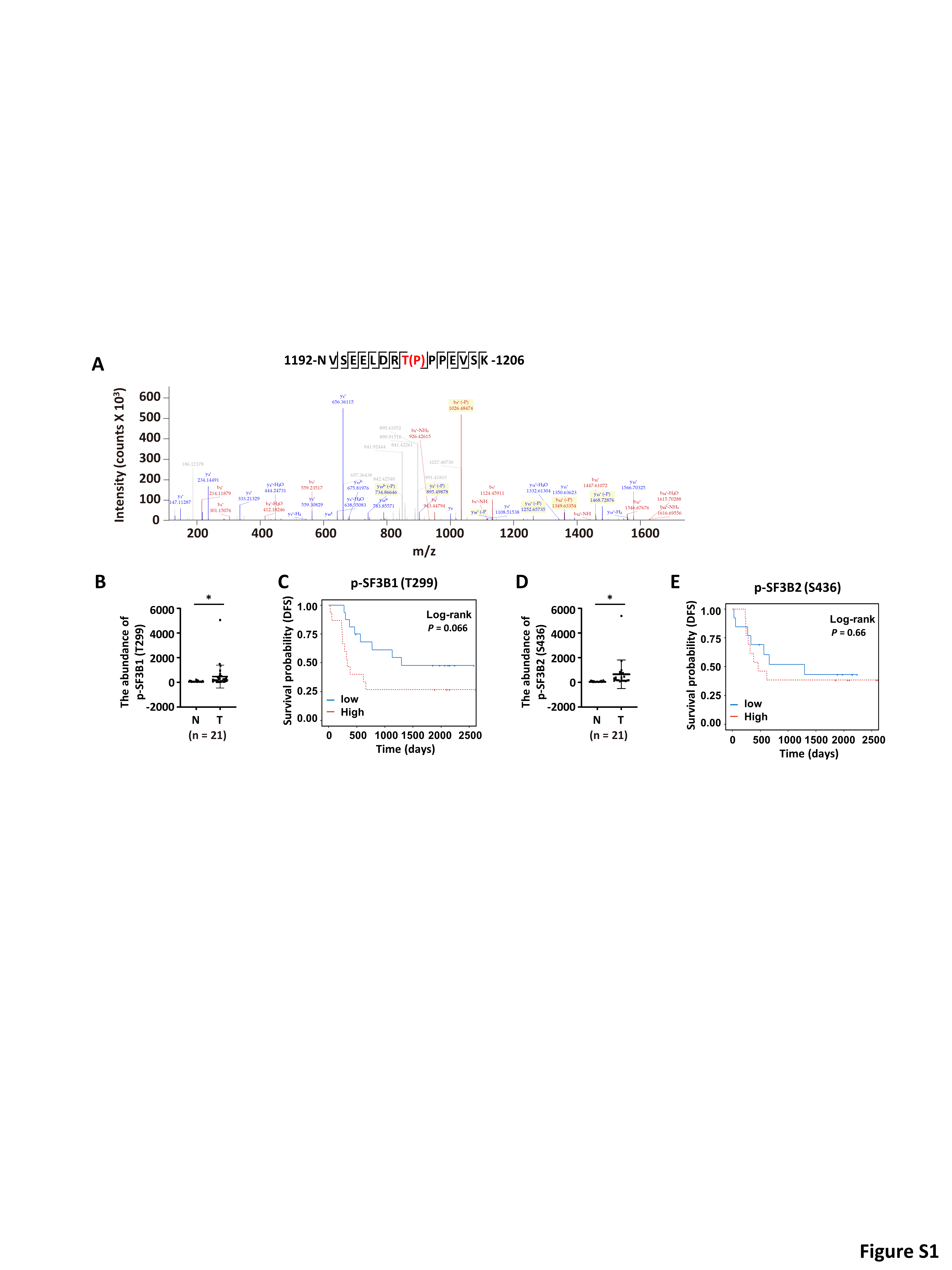

Supplement: S1 Fig — (A) Tandem MS spectrum of T1200 phosphorylation detected in SF3B3 is shown. (B, D) The abundance of p-SF3B1 (T299) (B) and p-SF3B2 (S436) (D) in ESCC tumor (T) and adjacent normal (N) tissues is shown (n = 21). (C, E) Kaplan–Meier plots of DFS in ESCC patients of p-SF3B1 (T299) (C) and p-SF3B2 (S436) (E). The data underlying the graphs shown can be found in S1 Data. (TIF) [file pbio.3003729.s001.tif]

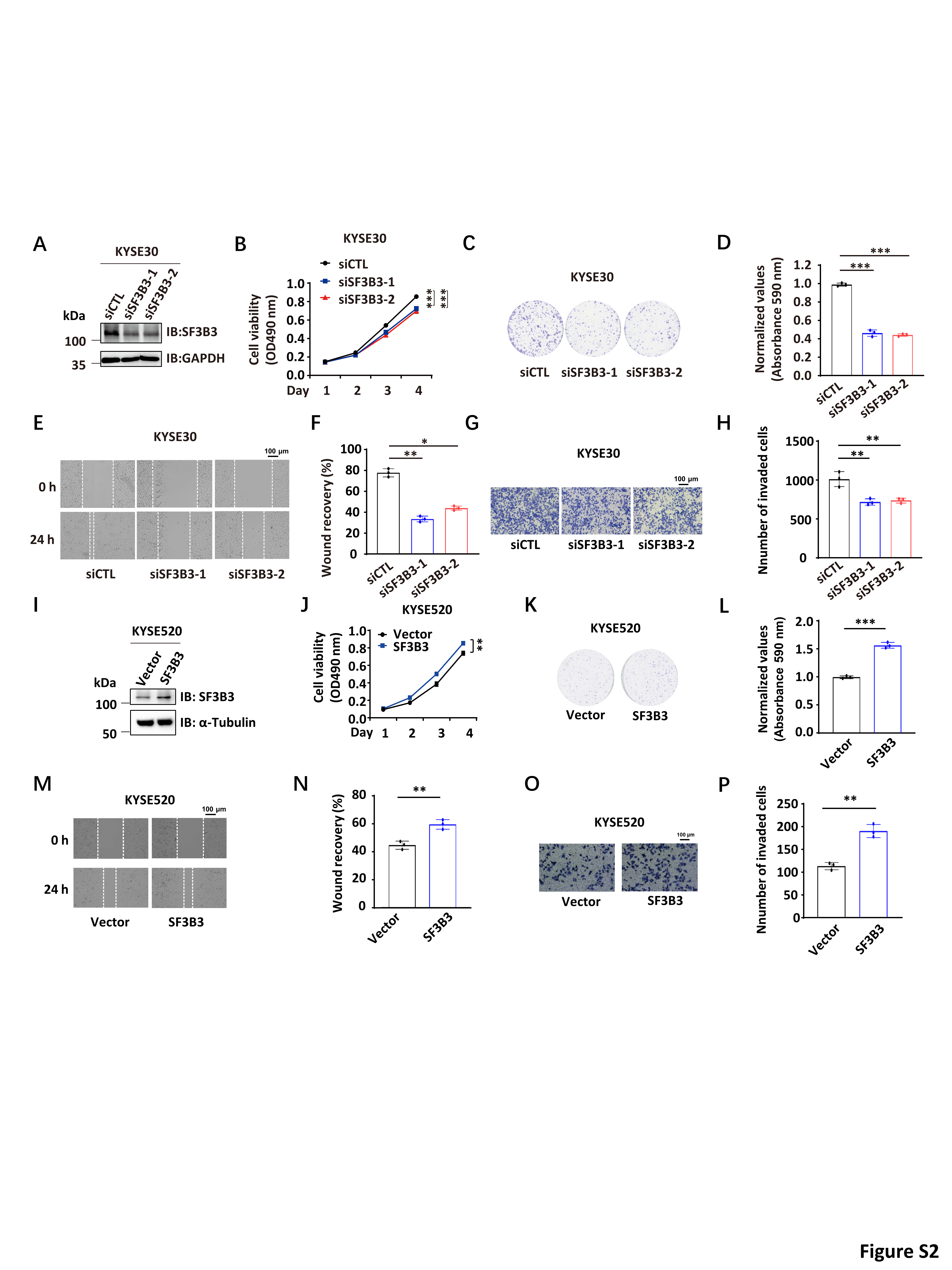

Supplement: S2 Fig — (A–C, E, G) KYSE30 cells were transfected with siCTL, siSF3B3-1, or siSF3B3-2, followed by IB analysis (A), cell proliferation assay (B), colony formation assay (C), wound healing assay (E), and transwell assay (G) (mean ± SD; ***P < 0.001, Student t test). (D) The quantification of the crystal violet dye in (C) is shown (mean ± SD; **P < 0.01, Student t test). (F) The quantification of the percentage of wound recovery in (E) is shown (mean ± SD; *P < 0.05, ***P < 0.001, Student t test). (H) The quantification of the number of invasive cells in (G) is shown (mean ± SD; ***P < 0.001, Student t test). (I–K, M, O) KYSE520 cells were infected with lentivirus expressing control vector or SF3B3 were subjected to IB analysis (I), cell proliferation assay (J), colony formation assay (K), wound healing assay (M), and transwell assay (O) (mean ± SD; **P < 0.01, Student t test). (L) The quantification of the crystal violet dye in (K) is shown (mean ± SD; ***P < 0.001, Student t test). (N) The quantification of the percentage of wound recovery in (M) is shown (mean ± SD; **P < 0.01, Student t test). (P) The quantification of the number of invasive cells in (O) is shown (mean ± SD; **P < 0.01, Student t test). The data underlying the graphs shown can be found in S1 Data. (TIF) [file pbio.3003729.s002.tif]

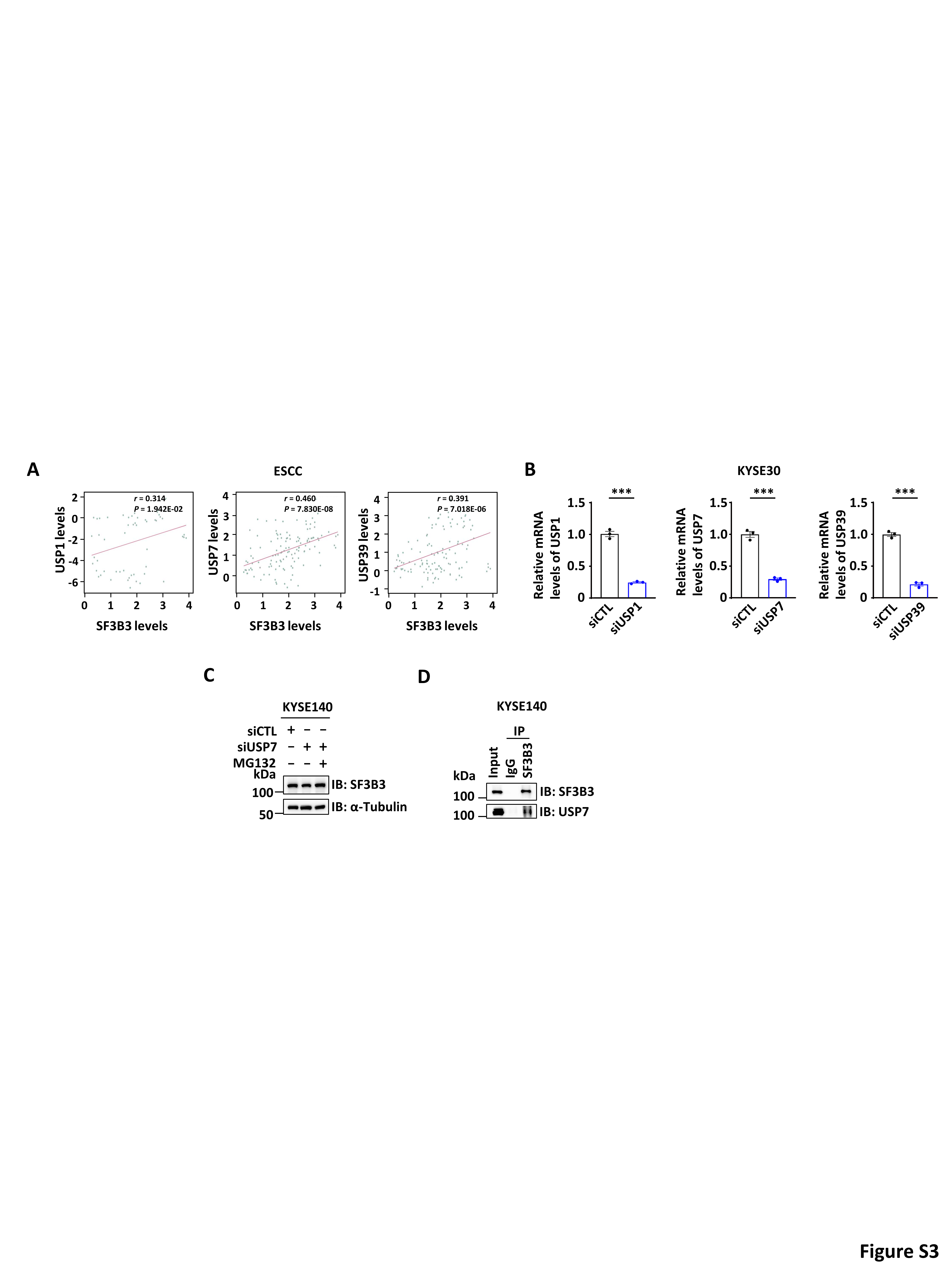

Supplement: S3 Fig — (A) The Pearson’s correlation of the expression levels between SF3B3 and USP1, USP7, or USP39 in ESCC in CPPA are shown. (B) KYSE30 cells transfected with siCTL, siUSP1, siUSP7, or siUSP39 for 48 h were subjected to RT-qPCR analysis to examine the mRNA levels of USP1, USP7, and USP39 (mean ± SD; *P < 0.05, **P < 0.01, ***P < 0.001, Student t test). (C) KYSE140 cells were transfected with siCTL or siUSP7 and then treated with or without the proteasome inhibitor MG132 (20 μM) for 10 h before IB analysis with antibodies as indicated. (D) KYSE140 cells were subjected to IP with control IgG or anti-SF3B3 antibody, followed by IB analysis with antibodies as indicated. The data underlying the graphs shown can be found in S1 Data. (TIF) [file pbio.3003729.s003.tif]

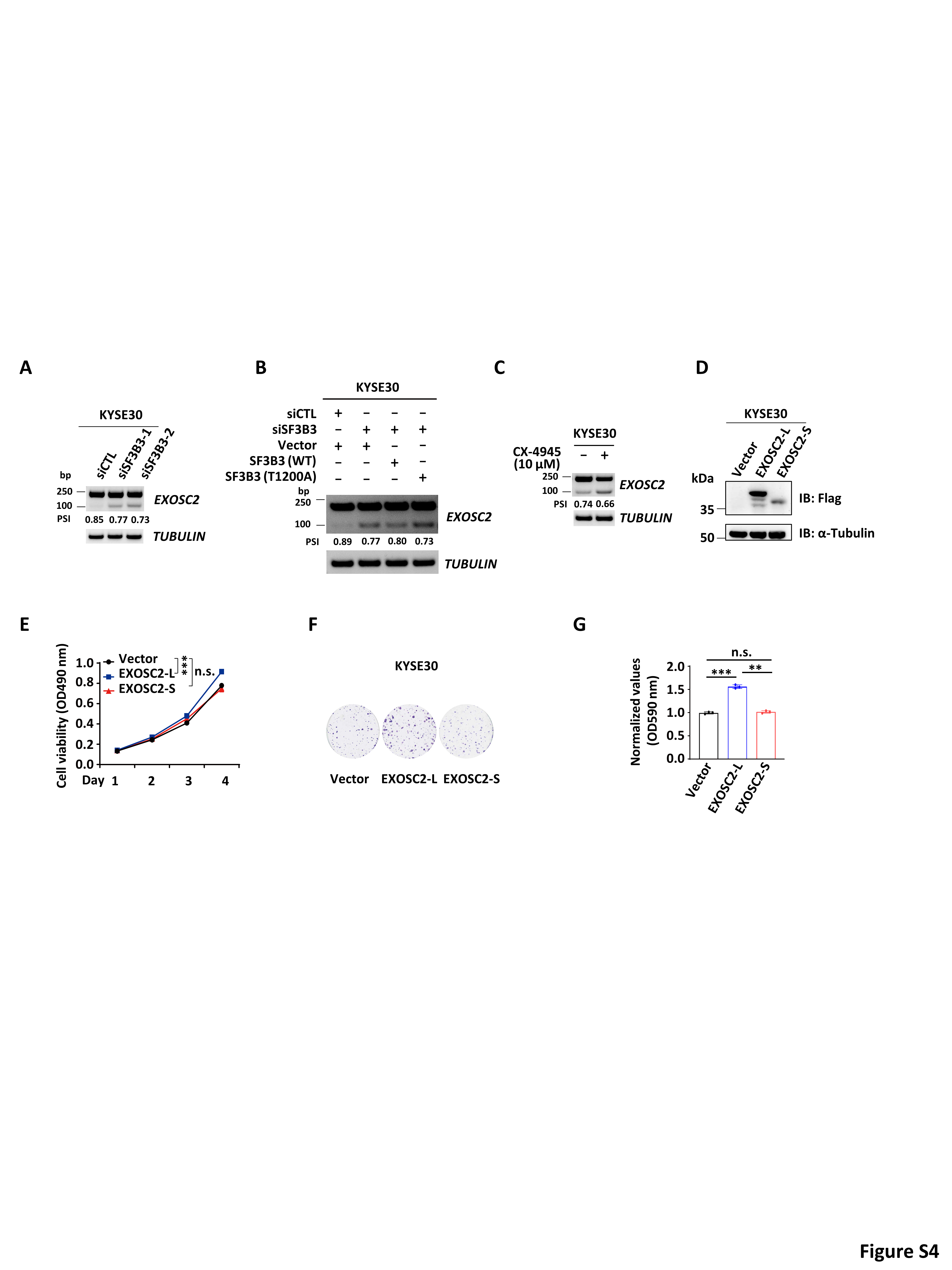

Supplement: S4 Fig — (A) KYSE30 cells transfected with siCTL, siSF3B3-1, or siSF3B3-2 were subjected to RT-PCR analysis to examine the expression of both short and long isoforms of EXOSC2 as indicated. PSI values were measured by Image J. (B) KYSE30 cells were transfected with siCTL or siSF3B3 in the presence or absence of control vector, SF3B3 (WT), or SF3B3 (T1200A), followed by standard PCR analysis. (C) KYSE30 cells treated with or without CX-4945 (10 μM) were subjected to standard PCR analysis to examine the alternative splicing of EXOSC2. (D–F) KYSE30 cells stably expressing control pCDH vector, EXOSC2-L, or EXOSC2-S were subjected to IB analysis (D), cell proliferation assay (E), and colony formation assay (F). (G) The quantification of the crystal violet dye in (F) is shown (mean ± SD; ns: not significant, **P < 0.01, ***P < 0.001, Student t test). The data underlying the graphs shown can be found in S1 Data. (TIF) [file pbio.3003729.s004.tif]

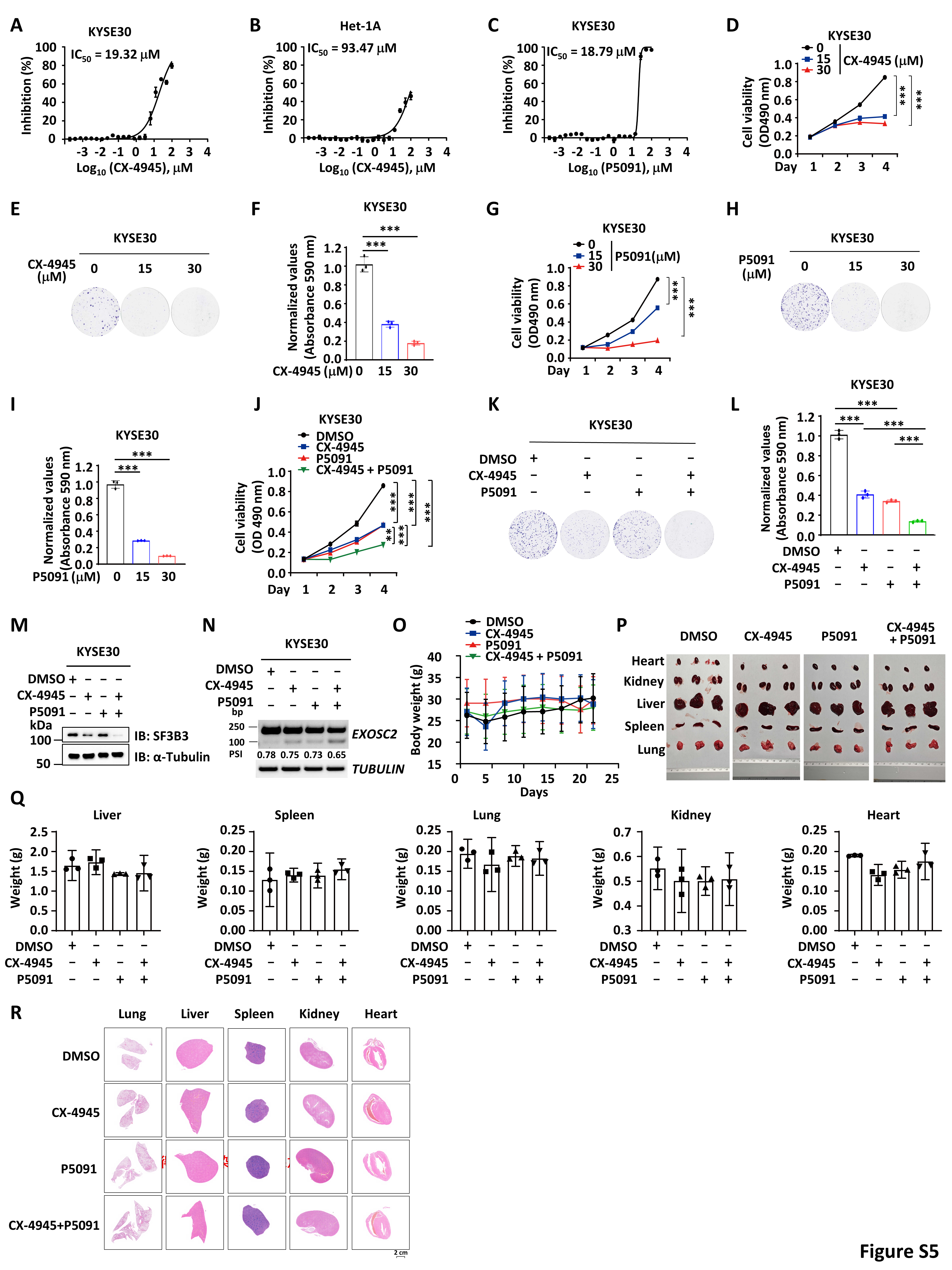

Supplement: S5 Fig — (A–C) KYSE30 and Het-1A cells were treated with CX-4945 (A, B) or P5091 (C) at concentrations as indicated for 72 h before cell viability measurement, and the IC50 are shown. (D, E) KYSE30 cells were treated with CX-4945 at concentrations as indicated, followed by cell proliferation (D) and colony formation (E) assay. (F) The quantification of the crystal violet dye in (E) is shown (mean ± SD; ***P < 0.001, Student t test). (G, H) KYSE30 cells were treated with P5091 at concentrations as indicated, followed by cell proliferation (G) and colony formation (H) assay. (I) The quantification of the crystal violet dye in (H) is shown (mean ± SD; ***P < 0.001, Student t test). (J, K) KYSE30 cells were treated with CX4945 (10 μM) and P5091 (10 μM) alone or in combination, followed by cell proliferation (J) and colony formation (K) assay. (L) The quantification of the crystal violet dye in (K) is shown (mean ± SD; ***P < 0.001, Student t test). (M, N) KYSE30 cells were treated with CX4945 (10 μM) and P5091 (20 μM) alone or in combination, followed by IB analysis (M) using antibodies as indicated and RT-PCR analysis (N) to examine the alternative splicing of EXOSC2. (O) The body weight of mice as described in Fig 7P is shown. (P) The organs as indicated from mice shown in Fig 7P are shown. (Q) The weight of the organs as described in (P) are shown. (R) The sections from organs as shown in (P) were subjected to hematoxylin and eosin (H&E) staining, and representative images are shown. The data underlying the graphs shown can be found in S1 Data. (TIF) [file pbio.3003729.s005.tif]
