# Supplementary material for: Casein kinase 2-mediated phosphorylation of the splicing factor SF3B3 plays a key role in esophageal squamous cell carcinoma progression
Source: PLoS Biol. 2026 Apr 10;24(4):e3003729. doi: 10.1371/journal.pbio.3003729 (PMC13068320; doi:10.1371/journal.pbio.3003729)
Supplement: S1 Raw Images — (PDF) [file pbio.3003729.s012.pdf]

Uncropped blots for Fig. 1G

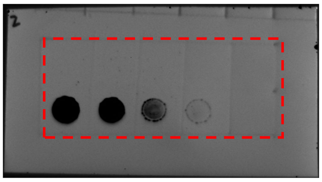

IB: p-SF3B3 (T1200)

Uncropped blots for Fig. 1H

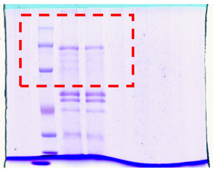

Uncropped blots for Fig. 1I

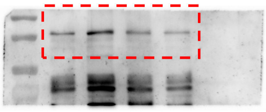

IB: p-SF3B3 (T1200)

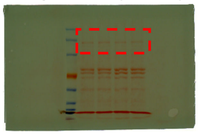

P.S

Uncropped blots for Fig. 1J

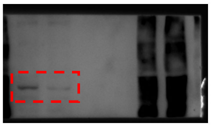

IB: p-SF3B3 (T1200)

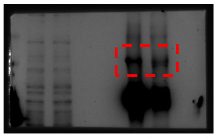

IB: p-SF3B3 (T1200)

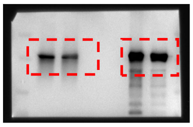

IB: SF3B3

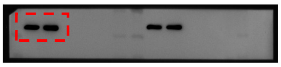

IB: α-Tubulin

Uncropped blots for Fig. 1K

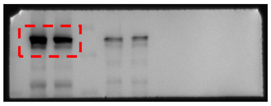

IB: GFP (Input)

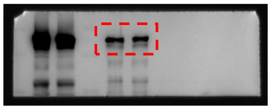

IB: GFP (IP: GFP)

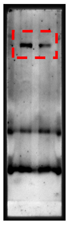

IB: p-SF3B3 (T1200)

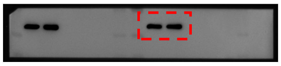

IB: α-Tubulin

Uncropped blots for Fig. 1L

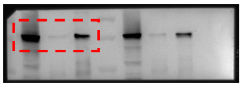

IB: SF3B3

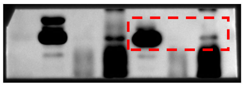

IB: CSNK2A1

Uncropped blots for Fig. 1M

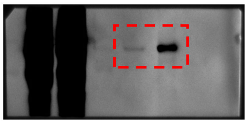

IB: GFP (IP: Flag)

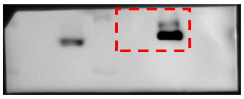

IB: Flag (IP: Flag)

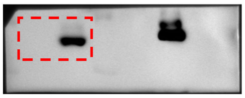

IB: Flag (Input)

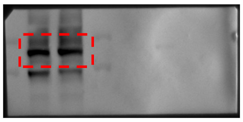

IB: GFP (Input)

Uncropped blots for Fig. 1N

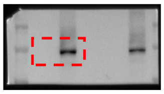

IB: Flag (Input)

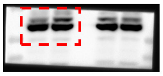

IB: Myc (Input)

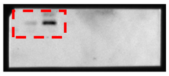

IB: Myc (IP: Flag)

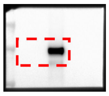

IB: Flag (IP: Flag)

Uncropped blots for Fig. 2A

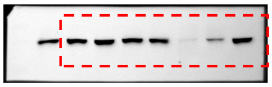

IB: SF3B3

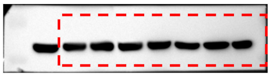

IB: α-Tubulin

Uncropped blots for Fig. 2B

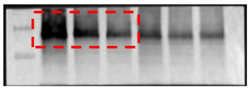

IB: SF3B3

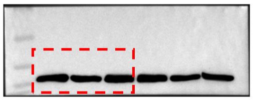

IB: GAPDH

Uncropped blots for Fig. 2J

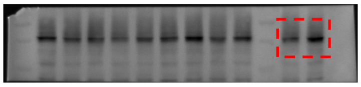

IB: SF3B3

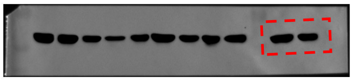

IB: α-Tubulin

Uncropped blots for Fig. 2R

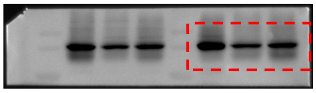

IB: SF3B3

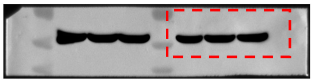

IB: α-Tubulin

Uncropped blots for Fig. 3A

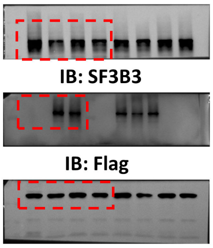

IB: SF3B3

IB: Flag

IB: α-Tubulin

Uncropped blots for Fig. 3E

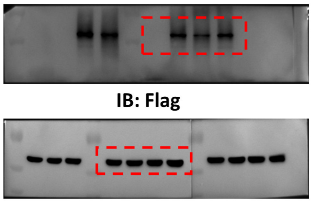

IB: Flag

IB: α-Tubulin

Uncropped blots for Fig. 4A

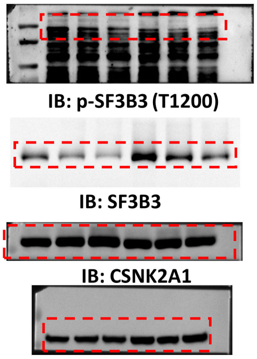

IB: p-SF3B3 (T1200)

IB: SF3B3

IB: CSNK2A1

IB: α-Tubulin

Uncropped blots for Fig. 4B

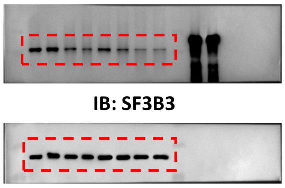

IB: SF3B3

IB: α-Tubulin

Uncropped blots for Fig. 4D

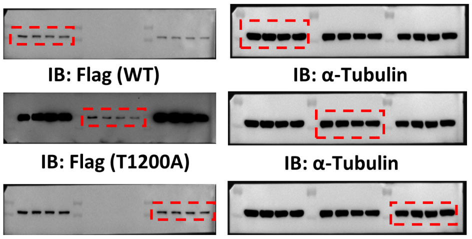

IB: Flag (WT)

IB: α-Tubulin

IB: Flag (T1200A)

IB: α-Tubulin

IB: Flag (T1200D)

IB: α-Tubulin

Uncropped blots for Fig. 5A

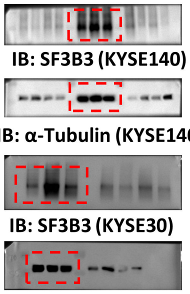

IB: SF3B3 (KYSE140)

IB: α-Tubulin (KYSE140)

IB: SF3B3 (KYSE30)

IB: α-Tubulin (KYSE30)

Uncropped blots for Fig. 5C

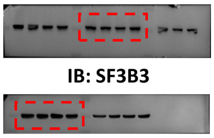

IB: SF3B3

IB: α-Tubulin

Uncropped blots for Fig. 5E

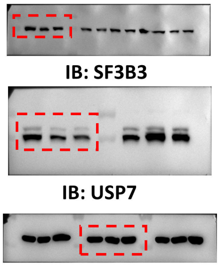

IB: SF3B3

IB: USP7

IB: α-Tubulin

Uncropped blots for Fig. 5F

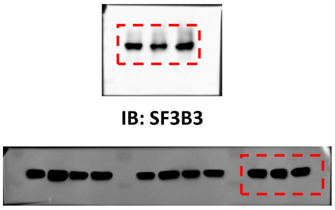

IB: SF3B3

IB: α-Tubulin

Uncropped blots for Fig. 5G

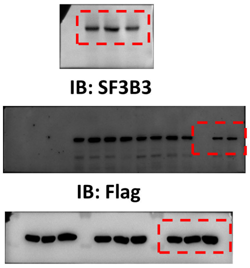

IB: SF3B3

IB: Flag

IB: α-Tubulin

Uncropped blots for Fig. 5H

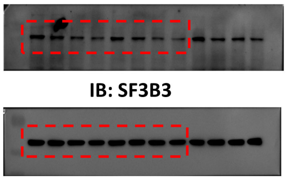

IB: SF3B3

IB: α-Tubulin

Uncropped blots for Fig. 5J

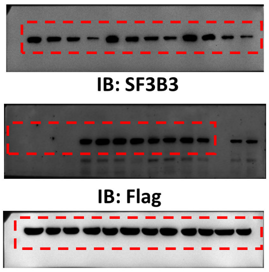

IB: SF3B3

IB: Flag

IB: α-Tubulin

Uncropped blots for Fig. 5L

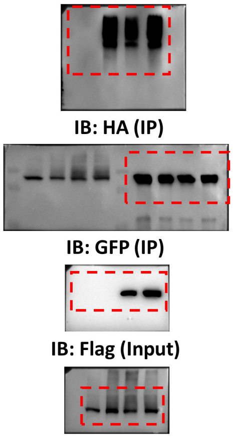

IB: HA (IP)

IB: GFP (IP)

IB: Flag (Input)

IB: GFP (Input)

Uncropped blots for Fig. 5M

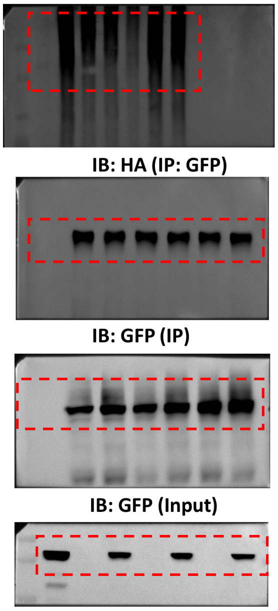

IB: HA (IP: GFP)

IB: GFP (IP)

IB: GFP (Input)

IB: Flag (Input)

Uncropped blots for Fig. 5N

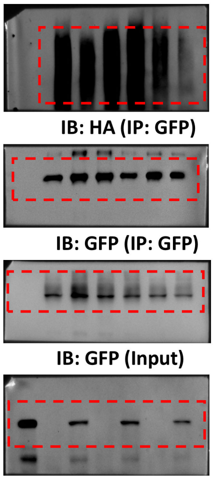

IB: HA (IP: GFP)

IB: GFP (IP: GFP)

IB: GFP (Input)

IB: Flag (Input)

Uncropped blots for Fig. 5O

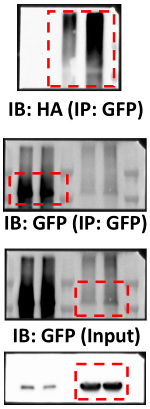

IB: HA (IP: GFP)

IB: GFP (IP: GFP)

IB: GFP (Input)

IB: α-Tubulin

Uncropped blots for Fig. 5P

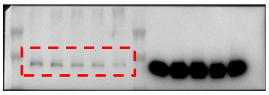

IB: SF3B3

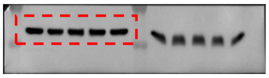

IB: α-Tubulin

Uncropped blots for Fig. 5Q

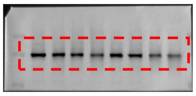

IB: SF3B3

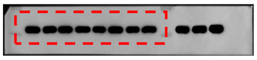

IB: α-Tubulin

Uncropped blots for Fig. 5S

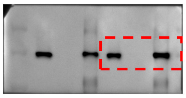

IB: SF3B3

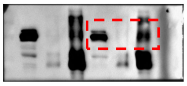

IB: USP7

Uncropped blots for Fig. 5T

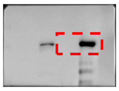

IB: Flag (IP: GFP)

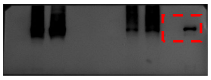

IB: GFP (IP: GFP)

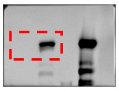

IB: Flag (Input)

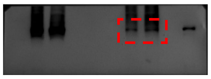

IB: GFP (Input)

Uncropped blots for Fig. 5U

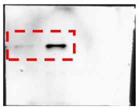

IB: Flag (IP: GFP)

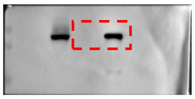

IB: GFP (IP: GFP)

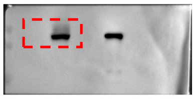

IB: GFP (Input)

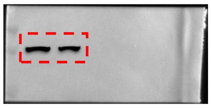

IB: Flag (Input)

Uncropped blots for Fig. 5W

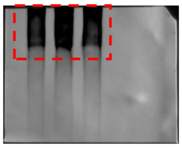

IB: HA

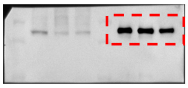

IB: GFP (IP: GFP)

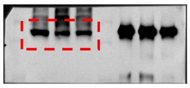

IB: GFP (Input)

Uncropped blots for Fig. 5X

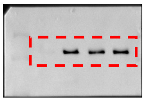

IB: Flag (IP: GFP)

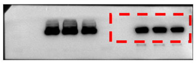

IB: GFP (IP: GFP)

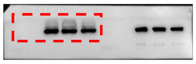

IB: GFP (Input)

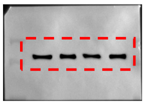

IB: Flag (Input)

Uncropped blots for Fig. 6G

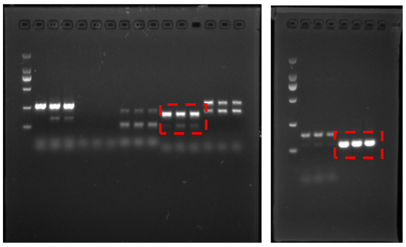

RT-PCR: *EXOSC2*

RT-PCR: *TUBULIN*

Uncropped blots for Fig. 6H

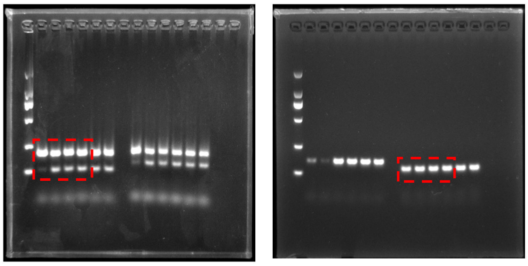

RT-PCR: *EXOSC2*

RT-PCR: *ACTIN*

Uncropped blots for Fig. 6I

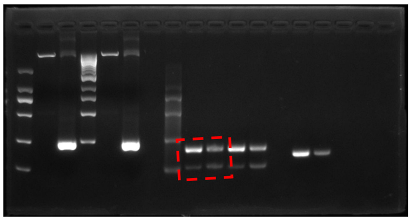

RT-PCR: *EXOSC2*

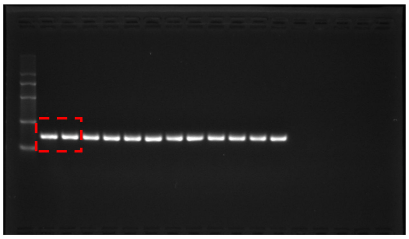

RT-PCR: *TUBULIN*

Uncropped blots for Fig. 6J

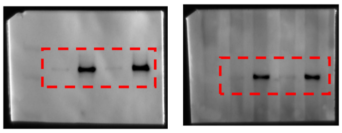

IB: SF3B1 (IP: GFP) IB: SF3B2 (IP: GFP)

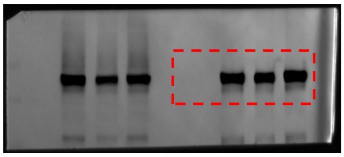

IB: GFP (IP: GFP)

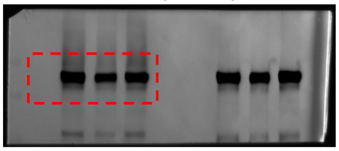

IB: GFP (Input: GFP)

Uncropped blots for Fig. 6L

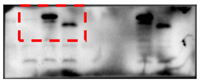

IB: Flag

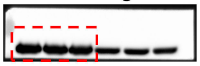

IB: α-Tubulin

Uncropped blots for Fig. 6P

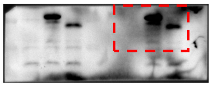

IB: Flag

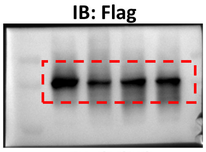

IB: SF3B3

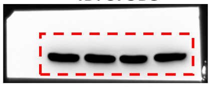

IB: α-Tubulin

Uncropped blots for Fig. 6W

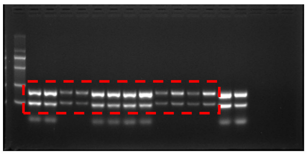

RT-PCR: *EXOSC2*

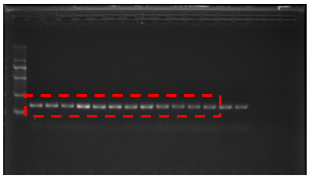

RT-PCR: *TUBULIN*

Uncropped blots for Fig. 7N

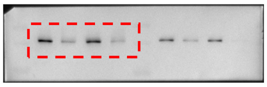

IB: SF3B3

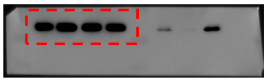

IB: α-Tubulin

Uncropped blots for Fig. 7O

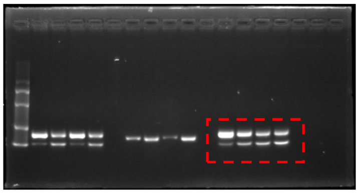

RT-PCR: *EXOSC2*

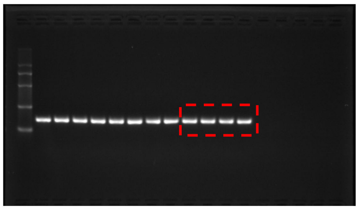

RT-PCR: *TUBULIN*

Uncropped blots for Fig. S2A

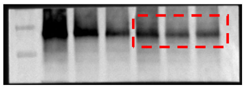

IB: SF3B3

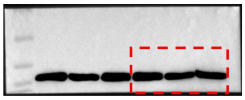

IB: GAPDH

Uncropped blots for Fig. S2I

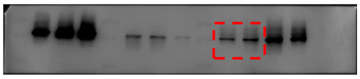

IB: SF3B3

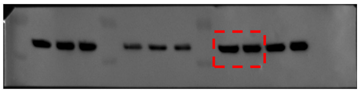

IB: α-Tubulin

Uncropped blots for Fig. S3C

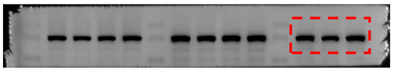

IB: SF3B3

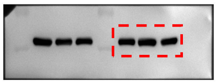

IB: α-Tubulin

Uncropped blots for Fig. S3D

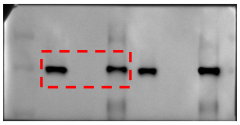

IB: SF3B3

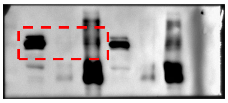

IB: USP7

Uncropped blots for Fig. S4A

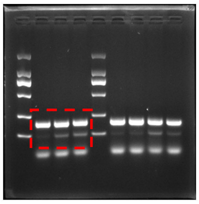

RT-PCR: *EXOSC2*

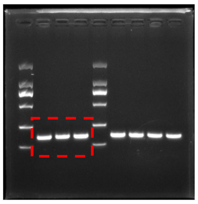

RT-PCR: *TUBULIN*

Uncropped blots for Fig. S4B

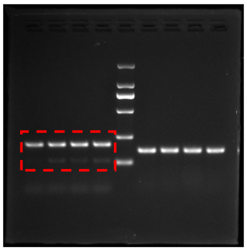

RT-PCR: *EXOSC2*

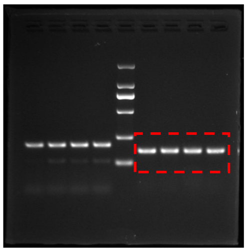

RT-PCR: *TUBULIN*

Uncropped blots for Fig. S4C

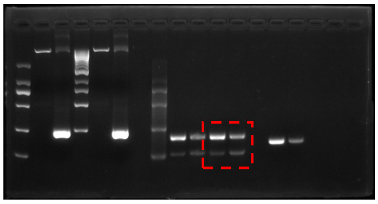

RT-PCR: *EXOSC2*

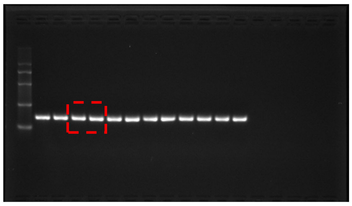

RT-PCR: *TUBULIN*

Uncropped blots for Fig. S4D

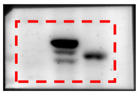

IB: Flag

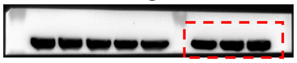

IB: α-Tubulin

Uncropped blots for Fig. S5M

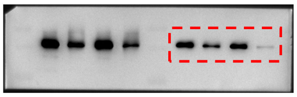

IB: SF3B3

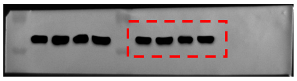

IB: α-Tubulin

Uncropped blots for Fig. S5N

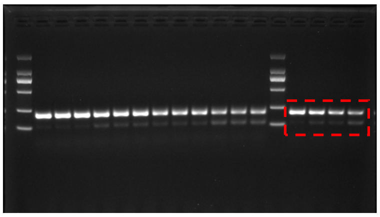

RT-PCR: *EXOSC2*

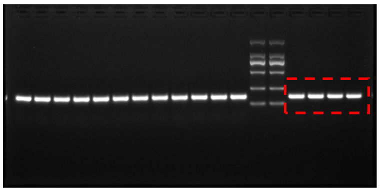

RT-PCR: *TUBULIN*
